# Ryanodine receptor leak triggers fiber Ca^2+^ redistribution to preserve force and elevate basal metabolism in skeletal muscle

**DOI:** 10.1126/sciadv.abi7166

**Published:** 2021-10-27

**Authors:** Cedric R. Lamboley, Luke Pearce, Crystal Seng, Aldo Meizoso-Huesca, Daniel P. Singh, Barnaby P. Frankish, Vikas Kaura, Harriet P. Lo, Charles Ferguson, Paul D. Allen, Philip M. Hopkins, Robert G. Parton, Robyn M. Murphy, Chris van der Poel, Christopher J. Barclay, Bradley S. Launikonis

**Affiliations:** 1 School of Biomedical Sciences, The University of Queensland, Brisbane, QLD 4072, Australia.; 2 Department of Biochemistry and Genetics, La Trobe Institute for Molecular Science, La Trobe University, Melbourne, VIC 3086, Australia.; 3 Leeds Institute of Medical Research, University of Leeds, Leeds, UK.; 4 Institute for Molecular Bioscience, The University of Queensland, Brisbane, QLD, Australia.; 5 Centre for Microscopy and Microanalysis, The University of Queensland, Brisbane, QLD, Australia.; 6 Department of Physiology Anatomy and Microbiology, La Trobe University, Melbourne, VIC 3086, Australia.

## Abstract

Muscle contraction depends on tightly regulated Ca^2+^ release. Aberrant Ca^2+^ leak through ryanodine receptor 1 (RyR1) on the sarcoplasmic reticulum (SR) membrane can lead to heatstroke and malignant hyperthermia (MH) susceptibility, as well as severe myopathy. However, the mechanism by which Ca^2+^ leak drives these pathologies is unknown. Here, we investigate the effects of four mouse genotypes with increasingly severe RyR1 leak in skeletal muscle fibers. We find that RyR1 Ca^2+^ leak initiates a cascade of events that cause precise redistribution of Ca^2+^ among the SR, cytoplasm, and mitochondria through altering the Ca^2+^ permeability of the transverse tubular system membrane. This redistribution of Ca^2+^ allows mice with moderate RyR1 leak to maintain normal function; however, severe RyR1 leak with *RYR1* mutations reduces the capacity to generate force. Our results reveal the mechanism underlying force preservation, increased ATP metabolism, and susceptibility to MH in individuals with gain-of-function *RYR1* mutations.

## Teaser

RyR1 Ca^2+^ leak causes a cascade of events that shifts Ca^2+^ to the cytoplasm and mitochondria, supporting force generation.

## INTRODUCTION

The sarcoplasmic reticulum (SR) is a highly specialized Ca^2+^ storage organelle within skeletal muscle cells that enables rapid coordinated release of Ca^2+^ to generate force in response to an action potential. In addition, adenosine 5′-triphosphate (ATP) hydrolysis by the SR Ca^2+^ adenosine triphosphatase (ATPase) (SERCA), which pumps cytoplasmic Ca^2+^ back into the SR following Ca^2+^ release, accounts for up to 15% of whole-body resting ATP use (*1*), which contributes to the heat generation necessary for maintenance of core body temperature. We hypothesize that the relationship between Ca^2+^ regulation and Ca^2+^ distribution in the SR, cytoplasm, and mitochondria is important not only for our understanding of how Ca^2+^ determines muscle force but also for our understanding of muscle energetics and its role in some causes of hyperthermia and myopathies.

Central to the control of cytoplasmic Ca^2+^ is ryanodine receptor 1 (RyR1), which forms a physical interaction with the voltage sensor of the transverse tubular (t-) system, allowing rapid transduction of excitation for precise and timely muscle contraction [excitation-contraction (EC) coupling] (*2*). Patients with specific variants in *RYR1* or genes encoding RyR1-associated proteins that make the SR leaky to Ca^2+^ are at risk of developing malignant hyperthermia (MH) under anesthesia in which volatile anesthetics trigger uncontrolled Ca^2+^ release and heat production (*3*). Excessive SR Ca^2+^ leak is also implicated in susceptibility to exertional heat illness (*4*), in which the cellular stress response resulting from sustained muscle activity may compound the SR Ca^2+^ leak.

Here, we investigate the mechanistic consequences of RyR1 Ca^2+^ leak and uncover a cascade of events that have implications for force and heat generation in skeletal muscle. We examine Ca^2+^ handling in muscle from a leaky RyR1 mouse model, which carries the gain-of-function p.G2435R variant that is isogenic to a human MH pathogenic variant (*5*). Both heterozygous (HET) and homozygous (HOM) *RYR1* knock-in (KI) mice live to adulthood (HOM mice have a reduced life span) but show a gene dose–dependent susceptibility to volatile anesthetics and increased ambient temperature. These triggers drive further Ca^2+^ leakiness and additive heat production. We further show that HET mice can generate normal force during EC coupling and thus resemble humans with the same and similar mutations, who usually lack any phenotype in the absence of external triggers (*3*). However, force generation in HOM mice is reduced by ~60%. We also studied mice lacking the major SR Ca^2+^ buffer calsequestrin 1 (CSQ1) (*6*), which display RyR1 Ca^2+^ leakiness and susceptibility to external triggers in the presence of normal RyR1 protein (*7*). Together, our results show that RyR1 Ca^2+^ leak triggers a cascade of events that precisely control the redistribution of intracellular Ca^2+^. This redistribution determines the capacity of the muscle to generate force, the basal turnover of ATP, and the conditions that increase susceptibility to hyperthermia induced by external triggers.

## RESULTS

### RyR1 Ca^2+^ leak increases with *RYR1* mutations and absence of CSQ1

To measure the extent of RyR1 leakiness and its effects on Ca^2+^ handling, we imaged extracellular Ca^2+^ in the t-system of skinned skeletal muscle fibers from eight groups: male and female wild-type (WT), HET, and HOM *RYR1* KI mice and CSQ1 null mice. HET and HOM *RYR1* KI mice provided an opportunity to investigate gene dose effects of Ca^2+^ leak, and CSQ1 null mice provided a model of RyR1 Ca^2+^ leak in the absence of *RYR1* mutation but lowered SR Ca^2+^ buffering capacity (*8*). Intact fibers were isolated from extensor digitorum longus (EDL) muscles and exposed to the Ca^2+^-sensitive dye rhod-5N for sufficient time to allow equilibration within the extensive t-system. Fibers were then mechanically skinned by peeling away the outer plasma membrane with fine forceps, causing the mouths of the t-system to seal over at their former interface with the outer plasma membrane, thereby trapping the membrane impermeant dye (*9*).

It is assumed that the [Ca^2+^] of the interstitial fluid is the same as that in the t-system of intact muscle, but no measurements have been made (*10*). The skinned fiber preparation has allowed calibrated measurements of [Ca^2+^]_t-sys_ and show [Ca^2+^]_t-sys_ is the same as the interstitial [Ca^2+^] (*11*, *12*). This indicates that Ca^2+^ in the interstitial fluid and t-system lumen are in equilibrium, as one would expect as ions can diffuse between the spaces in intact muscle but also that the t-system membrane is tuned to maintain [Ca^2+^]_t-sys_ throughout the fiber independently of ionic diffusion from the interstitial fluid. For our experimental approach, this shows that (i) the t-system membrane of the skinned fiber preparation continues to function physiologically, (ii) the skinned fiber allows measurements of [Ca^2+^]_t-sys_ that are inaccessible to intact fiber preparations, and (iii) the sealing of the t-system provides functional isolation of the t-system membrane Ca^2+^-handling properties from contamination by the slow diffusion of Ca^2+^ with the interstitial fluid. Therefore, the skinned fiber preparation could be used to measure the physiological properties of the t-system membrane to inform our understanding of the intact muscle physiology.

**Fig. 1.**
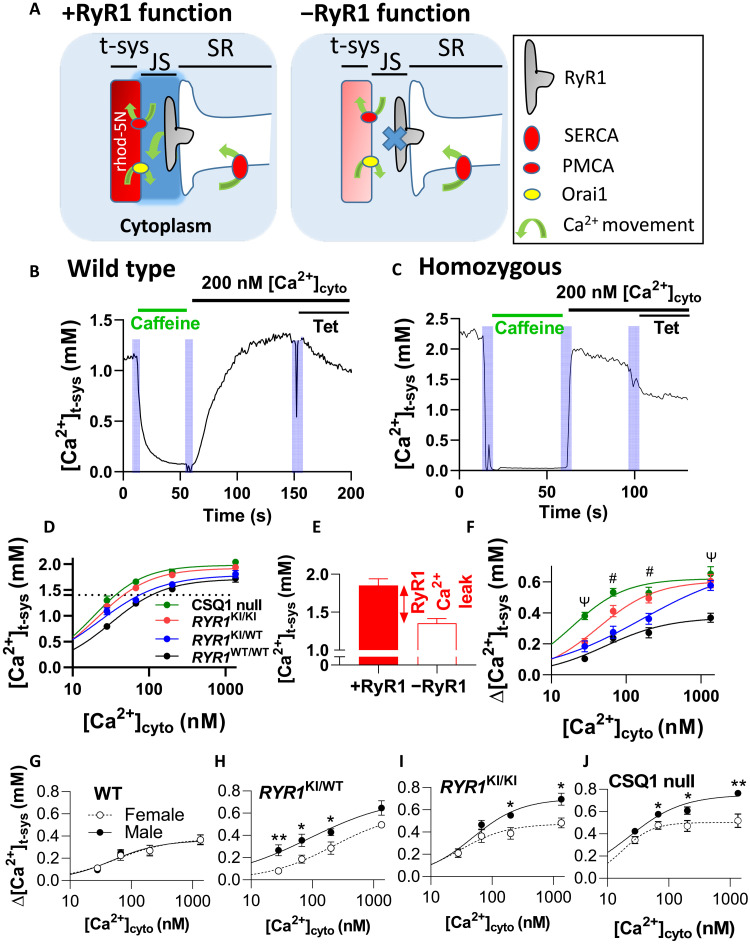
RyR1 Ca^2+^ leak increases with *RYR1* mutations and absence of CSQ1. (**A**) Schematic depicting the detection of RyR1 Ca^2+^ leak using rhod-5N trapped in the sealed t-system. The presence of functional RyR1 (+RyR1) allows Ca^2+^ to leak into the JS and increase [Ca^2+^]_JS_ above that of the bulk cytoplasm. [Ca^2+^]_JS_ directly influences PMCA activity and [Ca^2+^]_t-sys_. When RyR1 leak is blocked with Tet (−RyR1), [Ca^2+^]_t-sys_ drops. (**B**) [Ca^2+^]_t-sys_ (*t*) in a WT fiber and (**C**) in an HOM fiber during SOCE activation in caffeine, followed by caffeine washout and addition of standard solution with 200 nM [Ca^2+^]_cyto_ in the absence, then presence, of Tet. (**D**) Steady-state [Ca^2+^]_t-sys_ versus [Ca^2+^]_cyto_ in all genotypes. Intersection of dotted line and curves fitted to [Ca^2+^]_t-sys_ indicates the [Ca^2+^]_cyto_ where physiological [Ca^2+^]_t-sys_ is reached. (**E**) Example of the determination of Δ[Ca^2+^]_t-sys_ in +RyR1 and −RyR1 conditions in the presence of 200 nM [Ca^2+^]_cyto_ (HOM fiber). The difference between the histograms (Δ[Ca^2+^]_t-sys_) is RyR1 Ca^2+^ leak (text on panel). (**F**) RyR1 Ca^2+^ leak in each genotype. (**G** to **J**) RyR1 Ca^2+^ leak in genotypes by sex. Data are presented as means ± SEM and fitted by Hill curves. Statistics and *n* value details are in Materials and Methods. Two-way analysis of variance (ANOVA) [in (F)]: # = 4 significant results and Ψ = 3 significant results. Unpaired *t* test [in (G to J)]: *P < 0.05 and **P < 0.01. The *N* [in (D) and (F)] had a sex ratio of 1, and *n* [in (D) and (F)] had a sex ratio close to 1. In (G) to (J), *N* = 6 to 8 mice per genotype, *n* = 11 to 20 fibers per point with a sex ratio of approximately 1 for each.

Because RyR1 leakiness causes a local rise in [Ca^2+^] in the junctional space (JS) ([Ca^2+^]_JS_), the plasma membrane Ca^2+^ ATPase (PMCA) extrudes these ions across the t-system membrane, resulting in a rise in [Ca^2+^] in the sealed t-system ([Ca^2+^]_t-sys_) (Fig. 1A). Examples of changes in [Ca^2+^]_t-sys_ induced by cytoplasmic solutions reported by rhod-5N in WT and HOM fibers are shown (Fig. 1, B and C). In both examples, there is a reduction of [Ca^2+^]_t-sys_ during the activation of store-operated Ca^2+^ entry (SOCE) via SR Ca^2+^ depletion by caffeine, followed by establishment of millimolar-level steady-state [Ca^2+^]_t-sys_ by replacing caffeine solution with a standard solution containing 200 nM [Ca^2+^]_cyto_. The introduction of tetracaine (Tet) under identical ionic conditions reduces the [Ca^2+^]_t-sys_, showing the effect of blocking RyR Ca^2+^ leak. The use of Tet in the same fiber where RyR1 Ca^2+^ leak is being determined controls for differences in t-system Ca^2+^ handling between fibers and genotypes, which may have different populations of Ca^2+^ channels. Thus, the sealed t-system provides a nanodomain sensor of nanomolar changes in [Ca^2+^]_JS_, which in turn is set by RyR1 Ca^2+^ leak (*12*).

Steady-state [Ca^2+^]_t-sys_ following SR depletion and subsequent addition of different cytoplasmic [Ca^2+^] ([Ca^2+^]_cyto_) was determined for each genotype in the absence (Fig. 1D) and presence (fig. S1) of the RyR1 blocker Tet. Steady-state [Ca^2+^]_t-sys_ reached values between 1 and 2 mM as [Ca^2+^]_cyto_ was increased from 28 to 1342 nM (Fig. 1D; physiological [Ca^2+^]_t-sys_ indicated by a dotted horizontal line). Fitted Hill curves shifted progressively to the left as the number of *RYR1* mutations increased (from HET to HOM mice) and even further in the absence of CSQ1. Physiological levels of [Ca^2+^]_t-sys_ were reached at lower [Ca^2+^]_cyto_ as the severity of RyR1 leak increased, because [Ca^2+^]_JS_, and therefore the activity of t-system PMCAs, was higher in these mutants. To quantify RyR1 Ca^2+^ leak, we calculated the difference between steady-state [Ca^2+^]_t-sys_ with functional RyR1s and steady-state [Ca^2+^]_t-sys_ with blocked RyR1s (Δ[Ca^2+^]_t-sys_) (Fig. 1, E and F). Each data point was determined in the same fiber to improve the accuracy of this measurement (fig. S1). As expected, Δ[Ca^2+^]_t-sys_ (RyR1 Ca^2+^ leak) increased for a given [Ca^2+^]_cyto_ as the number of mutated *RYR1* alleles accumulated between WT, HET, and HOM *RYR1* KI mice (Fig. 1F). CSQ1 null mice displayed Δ[Ca^2+^]_t-sys_ values that were equal to or greater than those of HOM *RYR1* KI mice (Fig. 1F). While we acknowledge that the expression of RyR1 and dihydropyridine receptor (DHPR) is increased compared with the WT fibers (fig. S5), the relatively high RyR1 Ca^2+^ leak in CSQ1 null mice is a likely function of the reduced SR Ca^2+^ buffering capacity in the absence of CSQ1.

It has been reported that *RYR1* KI mice and CSQ1 null mice display a sex-specific susceptibility to volatile anesthetics and external heat stress. Male HOM *RYR1* KI (*5*) mice and male CSQ1 null mice (*6*) undergo spontaneous death more often than females in response to raised external temperature (*7*, *13*). To determine whether basal RyR1 Ca^2+^ leak forms the basis of this distinction, we analyzed the response of male and female mice within each genotype (Fig. 1, G to J). We could not resolve any difference in RyR1 leak between male and female WT mice, but we did resolve a clear separation of Δ[Ca^2+^]_t-sys_ at each [Ca^2+^]_cyto_ between male and female HET mice, such that males displayed greater RyR1 Ca^2+^ leak than females. In HOM *RYR1* KI and CSQ1 null mice, males displayed a greater RyR1 Ca^2+^ leak than females for [Ca^2+^]_cyto_ greater than 67 nM. Together, these results confirmed our ability to detect the severity of RyR1 Ca^2+^leak and that this leak is greater in male mice of each mutant genotype.

### RyR1 Ca^2+^ leak decreases SR Ca^2+^ content

**Fig. 2.**
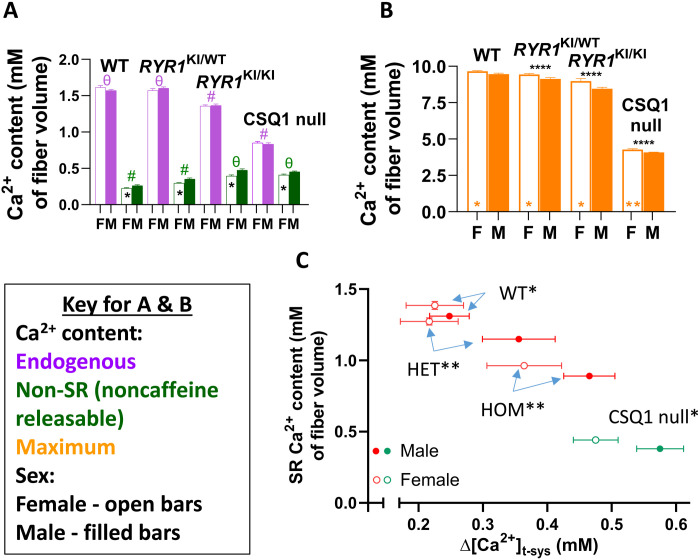
RyR1 Ca^2+^ leak decreases SR Ca^2+^ content. (**A**) Endogenous and non-SR (noncaffeine releasable) Ca^2+^ content of male (M) and female (F) *RYR1* KI and CSQ1 null fibers. (**B**) Maximum Ca^2+^ content of male (M) and female (F) *RYR1* KI and CSQ1 null fibers. (**C**) Endogenous SR Ca^2+^ content versus RyR1 Ca^2+^ leak in male and female *RYR1* KI and CSQ1 null fibers. Data from all graphs were compared using a pooled one-way ANOVA with Tukey’s multiple comparisons test to compare genotype. In (A), symbols denote # = 3 significant comparisons and θ = 2 significant comparisons. Significant differences between genotypes in each panel for endogenous and non–SR Ca^2+^ were found between WT and both HOM and CSQ1 null, HET and both HOM and CSQ1 null, as well as between HOM and CSQ1 null (all *P* < 0.0001). Sex differences in all graphs are represented by * inside the bars and were compared using an unpaired *t* test with Welch’s correction where *P < 0.05 and **P < 0.01. In (B), significant differences were found across all genotypes (above bars where ****P < 0.0001). Sex was compared within groups using an unpaired *t* test with Welch’s correction (bottom middle of bars). In (C), all pairwise comparisons between genotypes were significantly different (*P* < 0.0001). Data in all panels are shown as means ± SEM. Sample size: *N* = 4 to 8 mice per genotype; *n* = 8 to 13 fibers per point.

We next sought to determine the effect of RyR1 Ca^2+^ leak on the Ca^2+^ content of the SR. To do this, we determined the Ca^2+^ content of skinned fibers using a previously established membrane-lysis method, which measures the force response to Ca^2+^ liberated from lysed membrane compartments by a triton-oil emulsion [fig. S2; (*14*, *15*)]. Before submerging the fiber in the emulsion, the fiber was equilibrated in a cytoplasmic solution to either preserve or alter Ca^2+^ content within specific organelles. Equilibration for 2 min in a cytoplasmic bathing solution of 75 μM EGTA/100 nM Ca^2+^ minimized the net movement of Ca^2+^ across the SR and other membranes, so to allow determination of the total endogenous Ca^2+^ content that is set in vivo, minus the cytoplasmic Ca^2+^ that is replaced by the bathing solution. Therefore, it is not necessary to impose the reportedly higher [Ca^2+^]_cyto_ for some genotypes (*5*) to maintain the in vivo Ca^2+^ content (*14*, *15*). Bathing the fiber in a 30 mM caffeine/0.05 mM Mg^2+^ solution for 2 min opened the RyR1 to thoroughly deplete the SR of Ca^2+^, allowing the determination of the non–SR Ca^2+^ content. That is, the fiber Ca^2+^ remained trapped in the mitochondria, nuclei, golgi, and other non-SR compartments. The maximum Ca^2+^ holding capacity of skinned fibers was determined by fully loading the fiber with Ca^2+^ by bathing in a cytoplasmic solution containing 1 mM EGTA/200 nM Ca^2+^ for 4 min (*14*, *15*). Because the membranes must be lysed to assay Ca^2+^ content, a fiber can only be used for a single assay. Ca^2+^ content was measured in this way for each genotype and sex (Fig. 2, A and B). We found that endogenous Ca^2+^ in skinned fibers was equivalent in WT and HET mice but was successively lower in HOM and CSQ1 null mice (Fig. 2A). Non–SR Ca^2+^ content increased between WT, HET, and HOM/CSQ1 null mice (Fig. 2A). The maximal Ca^2+^ content of skinned fibers was only mildly decreased in HOM fibers, but considerably decreased in CSQ1 null fibers, as expected in the absence of the major Ca^2+^ storage buffer (Fig. 2B).

To calculate SR Ca^2+^ content, we computed the difference between measurements of total endogenous Ca^2+^ content and non–SR Ca^2+^ content in skinned fibers. When plotted as a function of Δ[Ca^2+^]_t-sys_, it became apparent that female WT muscle fibers had the greatest SR Ca^2+^ content and that this reduced as RyR1 Ca^2+^ leak increased (Fig. 2C). CSQ1 null mice showed a much lower SR Ca^2+^ content and departure from this relationship because of the significantly reduced SR Ca^2+^ buffering capacity of CSQ1 null muscle (*8*). Female mice maintained a higher SR content than male counterparts for each genotype, consistent with their less leaky RyR1s in each mutant genotype (Fig. 1, H to J). This also suggests the failure of the leak assay to detect an RyR1 leak difference between the sexes in WT (Fig. 1G) reflects a limit of this assay. These experiments revealed that Ca^2+^ leak through RyR1 causes a reduction of SR Ca^2+^ content and, in addition, that the SR contains less Ca^2+^ in male mice than in females.

### T-system Ca^2+^ permeability is dependent on chronic RyR1 Ca^2+^ leak

Resting [Ca^2+^]_cyto_ in intact muscle fibers has previously been shown to be higher in HET and HOM *RYR1* KI mice than in WT (*5*). However, the explanation for this chronic increase in [Ca^2+^]_cyto_ cannot be a simple redistribution of Ca^2+^ from the SR to the cytoplasm, as the t-system PMCA would lower [Ca^2+^]_cyto_ by extruding Ca^2+^ from the cytoplasm until [Ca^2+^]_cyto_ reached levels observed in WT muscle (*16*). This property means that persistently raised [Ca^2+^]_cyto_ must be due to changes in the Ca^2+^ handling properties of the plasma membrane—more Ca^2+^ must enter the fiber via leaking through the t-system membrane to account for persistently raised [Ca^2+^]_cyto_ in the presence of leaky RyR1s. Such a scenario would require a chronic increase in t-system Ca^2+^ permeability. Furthermore, to maintain a normal t-system Ca^2+^ gradient, an increased inward Ca^2+^ leak would need to be offset by an equivalent extrusion of Ca^2+^. That is, a physiological steady-state [Ca^2+^]_t-sys_ would be maintained by a “pump-leak” mechanism (Fig. 1D). Thus, increases in both t-system Ca^2+^ extrusion capacity and t-system Ca^2+^ leak are required to explain raised resting [Ca^2+^]_cyto_ during RyR1 Ca^2+^ leak (Fig. 1).

**Fig. 3.**
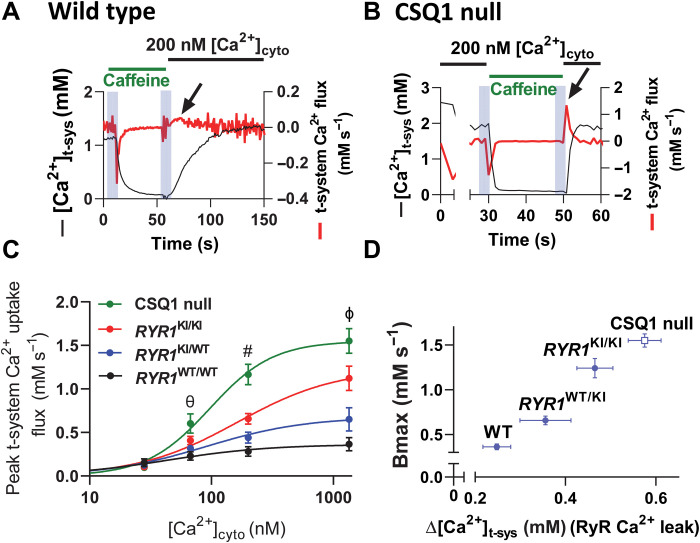
T-system Ca^2+^ permeability is dependent on chronic RyR1 Ca^2+^ leak. (**A**) [Ca^2+^]_t-sys_ (*t*) in a WT fiber during activation of SOCE via caffeine-dependent SR Ca^2+^ depletion, followed by establishment of steady-state [Ca^2+^]_t-sys_, in 200 nM [Ca^2+^]_cyto_ (black line). T-system Ca^2+^ flux is overlaid in red, and peak t-system Ca^2+^ extrusion flux is indicated by an arrow. (**B**) [Ca^2+^]_t-sys_ (*t*) and t-system Ca^2+^ flux in a CSQ1 null fiber using the same experimental protocol as in (A). Note the different scale of the right-hand *y* axes in (A) and (B). SOCE and t-system Ca^2+^ uptake are much more rapid events in CSQ1 null fibers than in WT. (**C**) Summary of peak t-system Ca^2+^ uptake rate in WT, HET, and HOM *RYR1* KI mice and CSQ1 null mouse fibers. (**D**) Bmax of peak t-system Ca^2+^ uptake plotted against the RyR1 Ca^2+^ leak determined for each genotype (from Fig. 1F). A two-way ANOVA with Tukey’s multiple comparisons was used to determine significant differences in peak t-system Ca^2+^ uptake flux by both genotype [*F*(3) = 122.06, *P* < 0.0001] and [Ca^2+^]_cyto_ [*F*(4) = 484.19, *P* < 0.0001]. There was also a significant difference of interaction between genotype and [Ca^2+^]_cyto_ [*F*(12) = 43.05, *P* < 0.0001]. Symbols represent the following: θ = 2 significant comparisons, # = 4 significant comparisons, and ϕ = 6 significant comparisons. 67 nM [Ca^2+^]_cyto_: WT and HET versus CSQ1, *P* < 0.0001, *P* < 0.001, respectively. 200 nM [Ca^2+^]_cyto_: WT versus Hom and CSQ1, *P* < 0.0001. HET versus HOM and CSQ1, *P* < 0.0001. 1342 nM [Ca^2+^]_cyto_: All genotypes are significantly different, *P* < 0.0001. *N* = 10 mice; *n* = 5 to 8 fibers per point.

We therefore determined the rate at which the t-system was able to pump (extrude) Ca^2+^ from the JS. We applied different [Ca^2+^]_cyto_ to Ca^2+^-depleted fibers while tracking transient changes in [Ca^2+^]_t-sys_ and multiplied the derivative of these transients by the Ca^2+^ buffering power of the t-system to determine t-system Ca^2+^ flux (Fig. 3, A and B) (*11*). First, the [Ca^2+^]_t-sys_ was depleted by exposure to caffeine to chronically activate SOCE. The peak Ca^2+^ extrusion rate (black arrows) occurred 1 to 5 s after the exchange of a caffeine solution for the standard solution. WT (Fig. 3A) and CSQ1 null (Fig. 3B) fibers displayed peak Ca^2+^ extrusion rates that differed by an order of magnitude. Similar experiments were performed for all genotypes across a range of [Ca^2+^]_cyto_. Peak extrusion rates were a function of [Ca^2+^]_cyto_ in the nanomolar to low micromolar range (Fig. 3C), consistent with Ca^2+^ being carried by the t-system PMCA (*12*, *17*). The maximal capacity of the t-system to extrude Ca^2+^ can be estimated from the Bmax of the curve fitted to peak t-system Ca^2+^ uptake versus [Ca^2+^]_cyto_, which increased as RyR1 Ca^2+^ leak increased (Fig. 3D). In the presence of increased RyR1 Ca^2+^ leak, we expect there to be higher [Ca^2+^]_JS_ (*12*), which may be the dependent variable that determines the t-system Ca^2+^ extrusion rate (*17*). Therefore, we checked the capacity of the t-system to extrude JS Ca^2+^ in the absence of RyR1 Ca^2+^ leak, where [Ca^2+^]_JS_ would be the same as that set in the cytoplasmic bathing solution. In WT and CSQ1 null muscle fibers, which represented the extremes of t-system Ca^2+^ permeability, the t-system peak Ca^2+^ uptake fluxes and the Bmax of the curves fitted to the data from WT and CSQ1 null fibers were significantly different (fig. S3). We conclude that RyR1 leakiness underlies the chronic increase in t-system Ca^2+^ extrusion capacity for each genotype. Furthermore, because there is a consistent steady-state [Ca^2+^]_t-sys_ because of pump-leak balance, the t-systems of WT, HET, HOM, and CSQ1 null mice must be progressively leakier. We confirmed this by monitoring the rate of divalent ion entry into myotubes derived from *RYR1* KI mouse muscle using Mn^2+^ quenching of cytoplasmic Fura-2. These experiments revealed that the rate of Mn^2+^ entry was increasingly sensitive to blocking RyR1 Ca^2+^ leak in the mutants in a gene dose–dependent manner (fig. S4). We did not explore t-system Ca^2+^ uptake rates for sex differences as our preliminary experiments indicated that the temporal resolution of our assay would not be sufficient to easily separate these groups within each genotype.

The increase in the capacity of the PMCA to extrude Ca^2+^ occurred without any change in PMCA protein expression in our two most extreme models (WT and CSQ1 null mice) (fig. S5). We can conclude that the apparent affinity of the PMCA for Ca^2+^ must be shifted to change the extrusion rate required to offset the influx of Ca^2+^ across the t-system of the resting fiber. Such a scenario could be explained by the binding of calmodulin to the PMCA, which is dependent on [Ca^2+^] (*17*, *18*). Together, our results reveal that RyR1 leak causes an increase in both t-system Ca^2+^ entry and Ca^2+^ extrusion.

### Increased [Ca^2+^]_cyto_ raises mitochondrial Ca^2+^ content

Having established that RyR1 leak increases [Ca^2+^]_cyto_ via changing the Ca^2+^ permeability of the transverse tubular membrane, we investigated the likely implications for the generation of heat in muscle (*19*–*21*). RyR1-mediated Ca^2+^ entry caused a reduction in the number of available cytoplasmic Ca^2+^-binding sites in muscles with leaky RyR1s because of a persistent increase in [Ca^2+^]_cyto_ (fig. S6, A and B). The raised [Ca^2+^]_cyto_ in turn caused an increase to the ATP consumption rate by SERCA and, consequently, increased heat output due to an increased metabolic requirement (fig. S6, C and D).

**Fig. 4.**
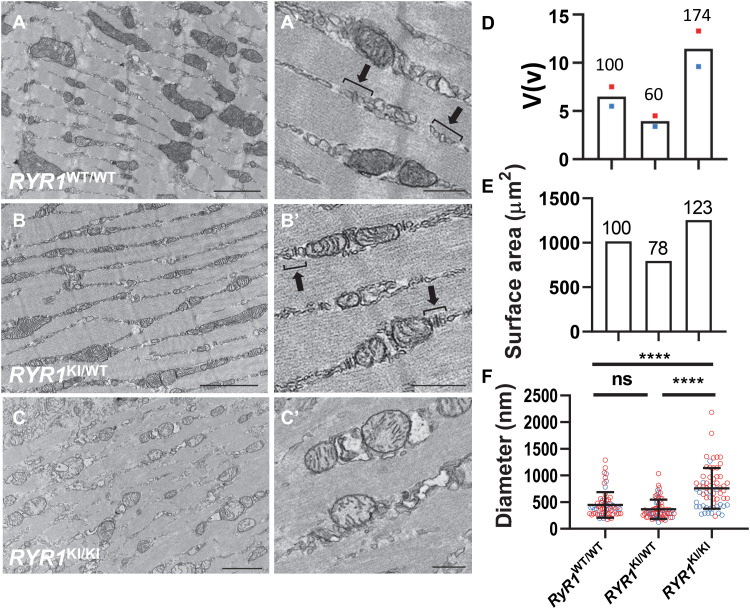
Mitochondrial morphology changes in *RYR1* KI mouse muscle. Ca^2+^ content. (**A**, **A′**, **B**, and **B′**) Electron micrographs of muscle from WT and HET, respectively, show normal sarcomeric and triad structure. (**C** and **C′**) Less defined triads and circular morphology of mitochondria in HOM. Images in (A′), (B′), and (C′) represent higher magnification images of areas in (A), (B), and (C) indicated by asterisk. Normal triad structures are denoted by parentheses in (A′) and (B′). Scale bars, 2 μm (A to C), 500 nm (A′ to C′). Mitochondrial ultrastructural parameters in WT, HET, and HOM were as follows: (**D**) Volume [as a % of cytoplasm, V(v)]. Relative volume to WT (in %) indicated above column. Colored dots are from male (blue) and female (red) mice, and column shows average value. (**E**) Surface area (μm^2^) calculated by measuring surface-to-volume ratio (Sv) multiplied by volume in (D). Relative surface area to WT (in %) indicated above column. (**F**) Mitochondrial diameter (nm; means ± SD, one-way ANOVA). Blue and red circles represent individual measurements from male and female mice. Statistical analysis was conducted across genotypes. ****P < 0.0001; ns, not significant.

It is possible that this persistent increase in basal metabolism resulted from increased [Ca^2+^]_cyto_ causing mitochondria to develop a greater capacity to produce ATP. Such a mechanism would be accompanied by changes in both mitochondrial morphology and Ca^2+^ content (*22*–*25*). Therefore, we next compared the ultrastructure of *RYR1* KI WT, HET, and HOM intact skeletal muscle by transmission electron microscopy (EM). Normal SR, t-system, and mitochondria structure were observed in WT and HET muscle (Fig. 4, A, A′, B, and B′, and fig. S7). However, in HOM muscle, the triad was less defined and the mitochondria appeared larger and more circular (Fig. 4, C and C′, and fig. S7). Stereological quantitation of mitochondrial volume density relative to cytoplasm, surface area, and mean diameter revealed that the total mitochondrial volume in the HOM muscle was increased, and this was associated with significantly larger mitochondrial profiles (Fig. 4, D to F). Little difference in the measured parameters between male and female mice was found, although a cluster of points from male mice for mitochondrial diameter was observed (Fig. 4F).

Enlargement of the mitochondria in CSQ1 null mice has been reported previously (*26*). We did not find any difference in the mitochondrial fusion protein mitofusin 2 (MFN2) between WT and CSQ1 null muscles. However, the mitochondrial-specific fission protein mitochondrial dynamics protein 49 (MiD49) was increased in CSQ1 null muscle, suggesting some dysregulation of mitochondrial dynamics in this genotype (fig. S8). We presume that the mitochondrial energy demands of the different genotypes underlie the morphological changes that we observed (*27*).

**Fig. 5.**
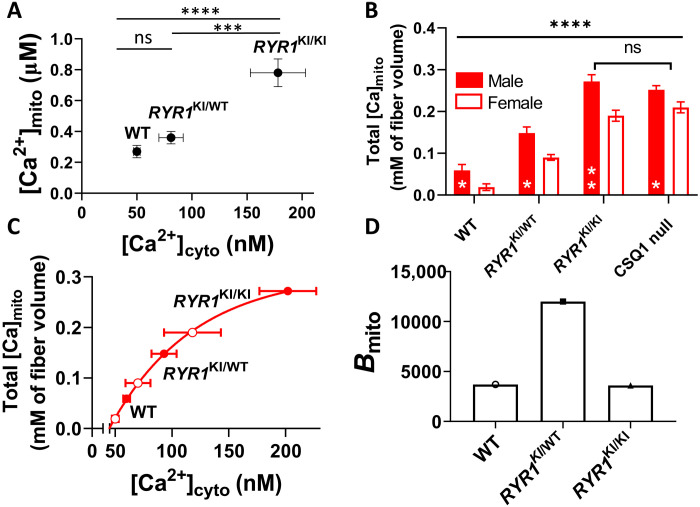
Increased [Ca^2+^]_cyto_ raises mitochondrial Ca^2+^ content. (**A**) Resting [Ca^2+^]_mito_ versus [Ca^2+^]_cyto_ in *RYR1* KI muscle. (**B**) Mitochondrial Ca^2+^ content (expressed per fiber volume) in muscle from male and female *RYR1* KI mice and CSQ1 null mice. Genotypes were compared using a pooled one-way ANOVA with Tukey’s multiple comparisons (*P* < 0.0001). All pairwise comparisons were significantly different (****P < 0.0001) except HOM and CSQ1 (ns). Sex was compared within groups using an unpaired *t* test with Welch’s correction, where *P < 0.05 and **P.0.01 (bottom middle of bars). (**C**) Mitochondrial Ca^2+^ content data from B plotted against the estimated resting [Ca^2+^]_cyto_ of intact fibers from female mice (see fig. S10). (**D**) Mitochondrial Ca^2+^ buffering power (*B*_mito_ = total Ca^2+^/free Ca^2+^) in *RYR1* KI mouse muscle. Data in (A) are presented as means ± SEM. Data in (B) are means ± SD. Sample size: *n* = 7, 9, and 4 for *RYR1*^WT/WT^, *RYR1*^KI/WT^, and *RYR1*^KI/KI^, respectively; statistics: one-way ANOVA. ****P < 0.0001; ***P = 0.0002. Mean resting [Ca^2+^]_cyto_ (±SD) of *RYR1* KI mouse muscle measured in intact fibers [data from (*5*)] was used as the dependent variable for mitochondrial Ca^2+^ in (A) to (C). For the purpose of this study, [Ca^2+^]_cyto_ of WT was normalized to 50 nM (*27*), and [Ca^2+^]_cyto_ values for mutants proportionally shifted to the left from that in (*5*).

To determine whether changes in [Ca^2+^]_cyto_ in the mutant mice affected Ca^2+^ levels in their mitochondria, we measured mitochondrial free [Ca^2+^] ([Ca^2+^]_mito_) in WT and mutant mouse muscles using rhod-2 and confocal microscopy (fig. S9). [Ca^2+^]_mito_ increased as a function of resting [Ca^2+^]_cyto_ in *RYR1* KI muscles (Fig. 5A), a consequence of which is reported to be modulation of the rate of ATP production (*25*). Mutant muscles should therefore have a greater capacity to resynthesize ATP, offsetting the likely increase in the rate of resting ATP consumption (fig. S6). We did not pursue measurements of [Ca^2+^]_mito_ between the sexes because of assay resolution limitations.

An increase in the total mitochondrial Ca^2+^ content is expected to underlie any increases in [Ca^2+^]_mito_. We suspected accumulation of mitochondrial Ca^2+^ following our measurements of a progressive increase in non-SR (noncaffeine releasable) Ca^2+^ content in mutated muscle fibers (Fig. 2A). However, the total Ca^2+^ content of skeletal muscle mitochondria has not previously been established. To determine how much of the non–SR Ca^2+^ is contained within mitochondria, we again used the membrane-lysis technique. Mitochondrial Ca^2+^ was depleted by abolishing the mitochondrial membrane potential with carbonyl cyanide *p*-trifluoromethoxyphenylhydrazone (FCCP) (figs. S2 and S10), and mitochondrial Ca^2+^ was calculated as the difference between the noncaffeine releasable Ca^2+^ content of fibers before and after mitochondrial Ca^2+^ depletion. These experiments revealed that total mitochondrial Ca^2+^ content progressively increased between WT, HET, HOM, and CSQ1 null mice. Furthermore, muscle fibers from male mice had a greater mitochondrial Ca^2+^ content than those from female mice (Fig. 5B). This increased mitochondrial Ca^2+^ content in mutant mice is consistent with their raised resting [Ca^2+^]_cyto_ (*5*). Although Lopez *et al*. (*5*) did not differentiate resting [Ca^2+^]_cyto_ between the sexes, an increase in [Ca^2+^]_cyto_ would be consistent with lower SR Ca^2+^ content and higher RyR1 leak in the males than females (Figs. 1 and 2). The increase in resting [Ca^2+^]_cyto_ provides a more favorable electrochemical gradient for Ca^2+^ to enter the mitochondria from the cytoplasm (*25*).

As we expected that [Ca^2+^]_cyto_ will be lower in females than in males for each *RYR1* genotype, we estimated the relationship between mitochondrial Ca^2+^ content and [Ca^2+^]_cyto_ in muscles from each of these mouse models. We constructed plots of male and female mitochondrial Ca^2+^ content against [Ca^2+^]_cyto_ (fig. S10) using [Ca^2+^]_cyto_ data available (*5*). Because these published data were not resolved by sex, we used the male data as reference and shifted the curve fitted to the female data to lower [Ca^2+^]_cyto_ to overlay the curve fitted to the male data points and set the [Ca^2+^]_cyto_ of female WT muscle to 50 nM [this reference value is not precisely known but is between 50 and 100 nM; (*28*–*30*)]. The data were well fitted by an exponential function, which revealed that mitochondria are sensitive to nanomolar levels of resting [Ca^2+^]_cyto_ (Fig. 5C) and that resting [Ca^2+^]_cyto_ in female and male CSQ1 null mice is about 125 and 170 nM (fig. S10), respectively.

The Ca^2+^ buffering power of an organelle determines its resting free [Ca^2+^] and the capacity to change this value as Ca^2+^ enters or leaves the organelle. Specifically, the Ca^2+^ buffering power of mitochondria (*B*_mito_) represents how many Ca^2+^ ions inside the organelle are bound for each one that is free (*B*_mito_ = [Ca^2+^ content]_mito_/free [Ca^2+^]_mito_, where Ca^2+^ is expressed per mitochondrial volume). *B*_mito_ is unknown in skeletal muscle; therefore, we estimated its value for WT and *RYR1* KI mice using the values determined in Figs. 4D and 5 (A and B). *B*_mito_ values increased significantly between WT (~3700) and HET (~12500) mice, but little change was observed between WT and HOM (~3600) mice. From the results in Figs. 4 and 5 and fig. S6, we conclude that RyR1 Ca^2+^ leak increases mitochondrial activity and, therefore, heat production at the muscle, as a result of increased [Ca^2+^]_cyto_, and [Ca^2+^]_mito_.

### Ca^2+^ redistribution preserves force generation

**Fig. 6.**
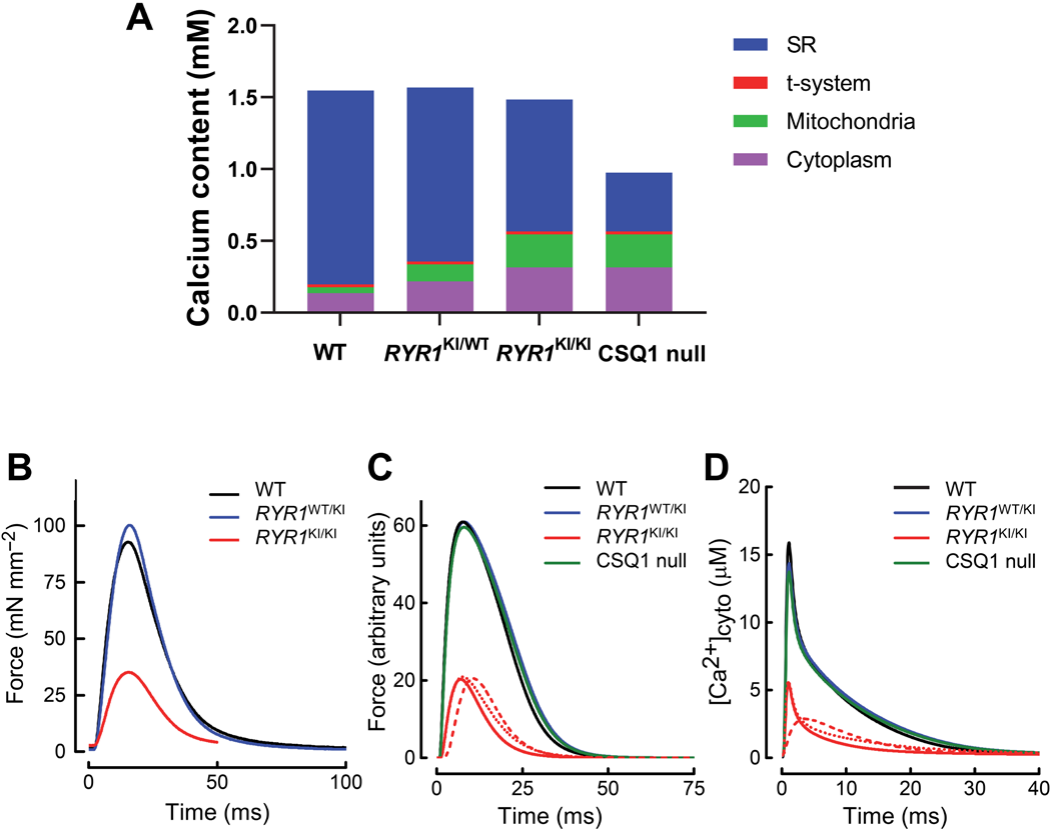
Ca^2+^ redistribution preserves force generation. (**A**) Ca^2+^ content (per fiber volume) of the major subcellular compartments in WT, HET, and HOM *RYR1* KI mice and CSQ1 null mouse muscle, shown as cumulative histograms (data from Figs. 2 and 4 and fig. S6). (**B**) Averaged records of force responses to a single stimulation of whole EDL muscle from WT (*n* = 4), HET (*n* = 7), and HOM (*n* = 4) *RYR1* KI animals. (**C**) Twitch force responses generated using our mathematical model. Ca^2+^ released in WT, HET, and HOM muscles was 300, 270, and 130 μM, respectively. The model underestimated the duration of relaxation in the HOM muscles (solid red trace); slower relaxation would occur if either the duration of the Ca^2+^ transient was increased (dashed line) or the SR Ca^2+^ pump was slower (dotted line). Note that all the modeled time courses are faster than those of the experimental records due to the absence of a series elastic component in the model, which slows twitch time course in muscles. (**D**) Modeled Ca^2+^ transients underlying the force responses in (C). The Ca^2+^ transient for HOM fibers is much smaller than those of the other genotypes, indicating impaired Ca^2+^ release. The dashed and dotted lines correspond to the use of prolonged Ca^2+^ release pulse and slower SR Ca^2+^ pump, as in (C).

The capacity of muscle to generate force during EC coupling will be affected by the redistribution of intracellular Ca^2+^ that we observed in muscle fibers from mice with leaky RyR1s (*20*, *21*). Visualization of this redistribution between SR, mitochondrial, and cytoplasmic compartments in fibers from *RYR1* KI mice revealed that total fiber Ca^2+^ content was relatively constant across the WT and *RYR1* KI muscles (Fig. 6A). However, as SR Ca^2+^ storage was progressively compromised by leaky RyR1s (WT < HET < HOM), the SR Ca^2+^ content reduced (blue bars). The very low SR Ca^2+^ content in CSQ1 null mice is due to the absence of CSQ1 buffering as well as RyR1 leak, preventing it from having comparable total fiber Ca^2+^ to the WT. The Ca^2+^ content of the cytoplasm (purple bars) and mitochondria (green bars) increased with progressive *RYR1* KI mutations, but was similar in HOM *RYR1* KI and CSQ1 null mice, despite the twofold difference in SR Ca^2+^ content. The RyR1 Ca^2+^ leak rate and resting [Ca^2+^]_cyto_ in HOM *RYR1* KI and CSQ1 null mice were similar (Figs. 1 and 4), consistent with [Ca^2+^]_cyto_ being the dependent variable determining mitochondrial Ca^2+^ content.

We measured force generation in EDL muscles from WT and *RYR1* KI mice to determine the effect of Ca^2+^ redistribution. Single twitch and tetanic force responses come from the hydrolysis of ATP that is quickly replenished by the creatine kinase in the cytoplasm. The absence of mitochondrial involvement in single force responses allows us to focus on the critical dependence of the amount and time course of Ca^2+^ release and thus as an indicator of RyR1 function and SR Ca^2+^ capacity. Average twitch force time courses from whole muscles revealed that peak twitch force was the same in WT and HET muscles, but each were significantly lower in HOM muscles (Fig. 6B). A similar result was found for maximal tetanic force across WT, HET, and HOM *RYR1* KI muscles (fig. S11). There were no significant differences in the measured times for twitch force to develop or relax (Table 1). Furthermore, the magnitude of tetanic force response could be maintained in both HET and HOM muscles (fig. S11), which contrasts with CSQ1 null muscle that cannot sustain force summation during trains of action potentials (*31*).

**Table 1.** Twitch force characteristics and model-derived parameters. *F*_tw_, maximum twitch force; *T*_c_, contraction time (time for force to increase from 1 to 100% *F*_tw_); *T*_R_, relaxation time (time for force to decrease from 90 to 10% *F*_tw_); SR leak rate, rate of Ca^2+^ efflux from SR in resting fiber (concentration expressed with respect to fiber volume); Ca^2+^ release, amount of Ca^2+^ released in response to single stimulus (concentration with respect to fiber volume); *Q*_Rest_, Ca^2+^-related rate of heat production in resting fiber.

	**Experimental data**				**Model-derived data**		
**Genotype**	***n***	***F*_tw_**	***T*_c_**	***T*_R_**	**Ca^2+^ release**	**SR leak rate**	***Q*_Rest_**
		kPa	ms	ms	μM	μM s^−1^	mW g^−1^
WT	4	100.9 ± 10.1	15.9 ± 0.6	35.0 ± 4.5	300	17.1	0.6
*RYR1* KI HET	7	101.3 ± 5.1	15.3 ± 0.4	26.5 ± 2.1	260	185.6	6.7
*RYR1* KI HOM	4	35.2 ± 12.2	15.4 ± 0.3	23.2 ± 1.2	130	261.7	9.4
CSQ1 null					220	238.5	8.5

By combining the redistribution of Ca^2+^ determined here (Fig. 6A) with our previously established mathematical model (*20*, *21*), we simulated twitch force and Ca^2+^ transients for each genotype (Fig. 6C). These simulations showed that the amount of Ca^2+^ released by a single stimulus must be graded across genotypes. That is, when resting [Ca^2+^]_cyto_ is high and intracellular Ca^2+^ buffers are more saturated, less Ca^2+^ release is needed to produce the same force response (e.g., compare WT and HET in Table 1). Previously, the CSQ1 null mouse was shown to generate twitch force equivalent to that of WT with a Ca^2+^ transient amplitude of only ~50% (*31*). We calculate that 200 μM Ca^2+^ is released during a single action potential stimulus in CSQ1 null muscle fibers. Because the raised resting [Ca^2+^]_cyto_ in these mice is likely to saturate cytoplasmic Ca^2+^-binding sites, this reduced Ca^2+^ release would support the capacity to induce a twitch force response that is indistinguishable from WT.

To infer the Ca^2+^ kinetics underlying twitch responses, we used our model to determine the amount of Ca^2+^ release required to mimic observed force responses in all mouse genotypes (fig. S12). Generally, the amount of Ca^2+^ released was inversely related to resting [Ca^2+^]_cyto_ (Table 1). That is, the greater the saturation of cytoplasmic Ca^2+^ buffers at rest, the less additional Ca^2+^ is required to produce a given twitch force. The exception to that scheme was the HOM *RYR1* KI genotype, for which the 40% less Ca^2+^ released by a single stimulus compared to WT fibers was associated with significantly reduced twitch force. The model analysis also predicted further disruption to Ca^2+^ handling in HOM fibers. If the only change to Ca^2+^ handling in these mice was reduced Ca^2+^ release, relaxation would be faster in HOM fibers than in WT or HET fibers. However, we observed the same rate of relaxation in HOM fibers as we did in other genotypes (compare solid red traces in Fig. 6, B and C). This could be explained by either slower removal of Ca^2+^ from the cytoplasm by SERCA (Fig. 6, C and D, dashed line) or a prolonged Ca^2+^ release pulse with a protracted decay phase (Fig. 6, C and D, dotted line). The calculated force traces assuming a prolonged Ca^2+^ release pulse bear closer resemblance to the measured force response, suggesting that this is a more likely scenario. Western blotting did not detect a difference in SERCA or RyR1 density between *RYR1* KI mice genotypes (fig. S13). Regardless, our model shows that HOM *RYR1* KI fibers release less Ca^2+^, during a prolonged timeframe, in response to a single stimulus.

Last, we used our model to estimate the rate of Ca^2+^ leak from the SR for each genotype (Table 1). The calculated rate of Ca^2+^ leak into the cytoplasm was 17.1 μM s^−1^ (relative to fiber volume) in WT fibers. Because [Ca^2+^]_cyto_ is constant in resting muscle, the leak of Ca^2+^ into the cytoplasm must be matched by the rate at which it is pumped back into the SR. The heat associated with the ATP splitting required to pump Ca^2+^ at 17.1 μM s^−1^ would be 0.6 mW (g whole muscle)^−1^, which is about 30% of the total heat production in resting mouse muscle (~2 mW g^−1^ at 22°C; (*32*)). Such a contribution is consistent with experimental data indicating that Ca^2+^ pumping accounts for between 30 and 50% of heat produced by resting mouse fibers (*33*, *34*). On the basis of our model, the magnitude of Ca^2+^ leak from the SR was greater in our mutant genotypes, and greatest in HOM and CSQ1 null fibers, for which we calculated a 15-times greater rate of Ca^2+^-related heat production than in WT fibers. In summary, although all mutant genotypes produced more heat, force generation was maintained by the precise redistribution of fiber Ca^2+^ in all except HOM *RYR1* KI fibers, where two mutated *RYR1* alleles are present.

## DISCUSSION

We have investigated a broad sample of effects from a spectrum of RyR1 Ca^2+^ leak in fast-twitch muscle fibers using WT, *RYR1* KI (HOM and HET), and CSQ1 null mice. We found that Ca^2+^ leak was dependent on the number of mutated *RYR1* alleles, sex, and SR Ca^2+^ buffering capacity. RyR1 Ca^2+^ leak triggered a precise redistribution of Ca^2+^ in fibers, while the total fiber Ca^2+^ content remained constant in *RYR1* KI compared with WT mice. We provide the first measures of mitochondrial Ca^2+^ content in muscle and show that this is sensitive to [Ca^2+^]_cyto_. The critical triggering event of fiber Ca^2+^ redistribution is RyR1 Ca^2+^ leak and Ca^2+^ entry across the transverse tubular membrane, setting [Ca^2+^]_cyto_. A functional consequence of the redistribution of fiber Ca^2+^ is to relieve the amount of Ca^2+^ release required for force generation during EC coupling. Specifically, in HET muscle fibers, the increase in cytoplasmic Ca^2+^ with lowered SR Ca^2+^ content maintained maximum force. In HOM fibers, redistribution of Ca^2+^ due to leaky RyR1s resulted in a further decrease in SR Ca^2+^ content and increase in [Ca^2+^]_cyto_ compared with HET. Compensation for the reduced SR capacity for Ca^2+^ release in HOM fibers was only partially offset by the basal loading of cytoplasmic Ca^2+^, suggesting that the RyR1 itself may have been too compromised to provide the Ca^2+^ release required for force generation like that observed in the WT.

The primary difference between the genotypes is RyR1 Ca^2+^ leak (Figs. 1 and 2), which proportionally activated a low amplitude resting Ca^2+^ influx. The influx may be activated by the depletion of the SR (SOCE), raised [Ca^2+^]_JS_, or potentially the activity of the PMCA. The most likely source of the influx is SOCE, which is a Ca^2+^ influx across the transverse tubular membrane, graded to the near-membrane depletion of SR terminal cisternae Ca^2+^ due to RyR1 activity (*11*, *12*, *35*). This is consistent with the levels of SR Ca^2+^ depletion and leakiness of the RyR1s across the genotypes (Figs. 1 and 2). The alternatives seem less likely. The presence of the fast Ca^2+^ buffer, 1,2-bis(2-aminophenoxy)ethane-N,N,N′,N′-tetraacetic acid (BAPTA), prevents the [Ca^2+^]_JS_ rise due to increasing RyR activity, but this intervention does not affect t-system Ca^2+^ entry activated from the SR (*35*). Furthermore, in the highest [Ca^2+^]_cyto_ applied in this study, [Ca^2+^]_t-sys_ rose or remained at its plateau (Fig. 1D). This is not consistent with increasing PMCA activity activating a t-system Ca^2+^ leak.

The redistribution of fiber Ca^2+^ requires a chronic change in the Ca^2+^ permeability of the t-system membrane (Fig. 3 and fig. S3). This is because the observed drop in SR Ca^2+^ content in proportion to RyR1 Ca^2+^ leak cannot be the direct source of the increased cytoplasmic or mitochondrial Ca^2+^ (*16*). Ca^2+^ must equilibrate across the t-system membrane. We measured a physiological [Ca^2+^]_t-sys_ and increasing t-system Ca^2+^ uptake capacity across all mutants (Figs. 1D and 3). This result can only be achieved if the t-system membrane maintains pump-leak balance. Thus, indicating that t-system resting Ca^2+^ influx, expected to be SOCE, must increase proportionately with Ca^2+^ extrusion capacity. Consistent with increasing SOCE with mutated *RYR1* alleles, our myotube studies showed a gene dose–dependent increase in resting Ca^2+^ influx that was sensitive to Tet (fig. S4).

Coordination of Ca^2+^ movements across the t-system membrane must be regulated by RyR1 leak. We suggest that RyR1 leak simultaneously generates Ca^2+^ nanogradients either side of the SR terminal cisternae membrane to do this. A near-membrane Ca^2+^ depletion and an increased [Ca^2+^]_JS_ would trigger low amplitude SOCE and chronically influence PMCA activity through Ca^2+^-calmodulin binding (*17*, *18*), respectively. The absence of change in PMCA density, presumably all at the surface membranes, across the mutants indicates that the affinity of the PMCA for Ca^2+^ must have changed (figs. S5 and S13).

We found that the females of each genotype had lower RyR1 Ca^2+^ leak, and therefore higher SR Ca^2+^ content than their male counterparts (Figs. 1 and 2). The greater stability of RyR1 in females allows the SR to be a stronger Ca^2+^ buffer than in male mice, providing female mice with an advantage in avoiding lethal hyperthermia during external stress (*5*, *7*, *13*, *36*). Consistent with RyR1 leak triggering t-system Ca^2+^ influx to increase [Ca^2+^]_cyto_ (Figs. 1 to 3 and fig. S4), blocking t-system Ca^2+^ channels in intact fibers of *RYR1* KI mice lowered resting [Ca^2+^]_cyto_ and partially reduced the halothane-induced [Ca^2+^]_cyto_ rise (*37*). Together, these results suggest that increasing resting cytoplasmic Ca^2+^ content is a factor in increasing susceptibility to MH.

### RyR1 leak and force

An increase in [Ca^2+^]_cyto_ in the presence of leaky RyR1s is essential for the ability of the muscle to produce normal force. Raised [Ca^2+^]_cyto_ in these mutant fibers caused more cytoplasmic Ca^2+^-binding sites to be presaturated with Ca^2+^ (fig. S6). In WT fibers, the first action potential in a train will trigger release of ~5 times more Ca^2+^ than subsequent action potentials (*19*, *20*, *38*, *39*). The need for the initially large release of Ca^2+^ from the SR is to saturate the cytoplasmic Ca^2+^-binding sites (*20*, *38*). A single action potential in the HET or CSQ1 null muscle caused the release of 10 and 50% less Ca^2+^ than in WT, respectively. This reduced SR Ca^2+^ release helps mutant fibers to maintain their SR Ca^2+^ load to support further Ca^2+^ release. Without the compensation provided by basally raised [Ca^2+^]_cyto_ to preload cytoplasmic Ca^2+^-binding sites in HET *RYR1* KI and CSQ1 null fibers, the reduced SR Ca^2+^ content and lower SR Ca^2+^ release of the mutants would result in reduced force generation compared with WT (*39*).

The reduced capacity of the HOM muscle to produce twitch and maximal tetanic force compared with the CSQ1 null, despite the similar levels of cytoplasmic Ca^2+^ and greater SR Ca^2+^ content of the HOM, indicates functional defects in the RyR1, preventing adequate Ca^2+^ release in HOM muscle (Figs. 1, 2, and 6). However, the ability of HOM muscle to maintain its maximum force during a tetanic contraction (fig. S9), in contrast to the inability of CSQ1 null muscle to do this (*31*), indicates the importance of CSQ1 and the SR capacity in sustaining Ca^2+^ release trains.

### RyR1 leak and mitochondria

A consequence of raised basal [Ca^2+^]_cyto_ is increased mitochondrial Ca^2+^ (Fig. 5) and a small but constant increased turnover of ATP by SERCA as Ca^2+^ continuously cycles through the leaky SR [fig. S6; Table 1; (*19*)]. The increased basal demand for ATP resynthesis is supported by the increase in [Ca^2+^]_mito_ (Fig. 5), which modulates ATP production (*24*, *25*). This loop of increased ATP use and resynthesis triggered by raised [Ca^2+^]_cyto_, shown in our cross-sectional study of mutant mice, may be a mechanism that can alter mitochondrial function and morphology in healthy muscle if RyR leak is triggered in a transient or intermittent manner by exercise (*40*, *41*).

This study has provided the first measurements of free and total Ca^2+^ inside the mitochondria of muscle fibers under different degrees of RyR Ca^2+^ leak. When expressed per fiber volume, mitochondrial Ca^2+^ content increased in an apparently simple way with a chronic increase in [Ca^2+^]_cyto_ across the genotypes and sex. *B*_mito_ increased in the HET compared with the WT, but *B*_mito_ was similar in the WT and HOM. A factor in the apparently abrupt changes in *B*_mito_ may be the different mitochondrial volumes displayed by the genotypes. We cannot be sure whether the spatial distribution of Ca^2+^ within the mitochondria of the different muscle genotypes remains the same or not, which would affect the calculation of *B*_mito_. Alternatively, the changing *B*_mito_ may be a function of the stress induced by raised resting [Ca^2+^]_cyto_, basal ATP use, and mitochondrial morphology [Figs. 4 and 5; (*22*, *27*)] affecting the dynamic amorphous Ca^2+^ phosphate formation that buffers mitochondrial Ca^2+^ (*42*, *43*). Regardless, in each genotype, *B*_mito_ is large, which will suppress changes in [Ca^2+^]_mito_ during EC coupling to the nanomolar range (*44*) and will also slow the exit of Ca^2+^ from mitochondria. The latter property would likely cause Ca^2+^ accumulation in the mitochondria during prolonged muscle activity.

*RYR1* mutations underlie susceptibility to MH and exertional heat stroke but also myopathies such as central core disease (CCD), which cause muscle weakness (*45*). Our results show the cascade of events stemming from increased RyR1 Ca^2+^ leak that will cause Ca^2+^ redistribution and possible changes in mitochondrial morphology in muscle of patients with CCD.

Our study describes the cost-benefit of regulated redistribution of Ca^2+^ in the presence of leaky RyR1s, so that force generation is not compromised by a single mutated *RYR1* allele. The cost of this adaptation is basal metabolic stress and increased susceptibility to hypermetabolism. The further increase in RyR1 leakiness in the HOM muscle caused a more extreme redistribution of fiber Ca^2+^ than the HET, but this could not completely rescue twitch force generation, as action potential stimulation of Ca^2+^ release was reduced and protracted. However, the HOM mouse used in our study could survive to adulthood (with a reduced life expectancy compared with WT) with the increased level of metabolic stress and reduced muscle force capacity. Our results thus reveal that, in healthy muscle, a stable RyR1 provides the SR with maximum Ca^2+^ storage capacity and a cytoplasm that is relatively free of Ca^2+^. This provides the muscle with not only optimal force generation capacity but also a defense against external stress–induced heatstroke or hyperthermia. The free cytoplasmic Ca^2+^-binding sites in resting muscle are a “safety net,” providing buffering sites for Ca^2+^ during uncontrolled release of SR Ca^2+^ that limit the increase in [Ca^2+^]_cyto_ and ATP consumption.

## MATERIALS AND METHODS

All experimental methods using mice were approved by the Animal Ethics Committees at The University of Queensland and at La Trobe University. Male and female C57/Bl6 mice WT, HET, and HOM for the p.G2435R variant of *RYR1* (*5*) and 2- to 6-month-old CSQ1 null (*6*) mice were euthanized by asphyxiation via CO_2_ exposure, and the EDL muscles were rapidly excised from the animals. Muscles were then placed in a petri dish under paraffin oil above a layer of Sylgard. Muscles were either mechanically skinned for force measurements or Ca^2+^ imaging (see below) or frozen for later Western blot analysis or EM (see below).

### Measuring RyR Ca^2+^ leak

Rhod-5N salt was trapped in the sealed t-system as originally described (*9*). Briefly, small bundles of fibers from EDL muscles were isolated using fine forceps and exposed to a “dye solution” applied through a microcap pipette that contained NaCl, 145 mM; KCl, 3 mM; CaCl_2_, 2.5 mM; MgCl_2_, 2 mM; fluo-5N salt, 1 mM; or rhod-5N salt, 2.5 to 7.5 mM; and Hepes, 10 mM (pH adjusted to 7.4 with NaOH). The dye solution was allowed more than 10 min to diffuse into the t-system from the surrounding bubble of solution. After this equilibration period, individual fibers that had been exposed to the solution were isolated and mechanically skinned. The final [rhod-5N] in the t-system was expected to be in the micromolar (*11*, *35*). After skinning, fibers were transferred to an experimental chamber containing a K^+^-based internal solution that allowed the sealed t-system to generate a normal resting membrane potential (*46*). The cytoplasmic solution was composed of EGTA, 50 mM; ATP, 8 mM; creatine phosphate, 10 mM; Hepes, 90 mM; Na^+^, 36 mM; K^+^, 126 mM; Mg^2+^, 1 mM; and *n*-benzyl-*p*-toluene sulphonamide, 0.05 mM. [Ca^2+^] in the cytoplasmic solution was set at either 0, 28, 67, 200, or 1342 nM. To thoroughly deplete Ca^2+^ from the SR, the fiber was exposed to a release solution similar to the cytoplasmic solution, except where [Mg^2+^] was lowered from 1 to 0.01 mM and 30 mM caffeine was added. [Ca^2+^] was nominally 0 in this solution.

Mounted skinned fibers were imaged using an Olympus FV1000 confocal microscope equipped with a 0.9 numerical aperture (NA) 40× Plan-Apochromat objective. Rhod-5N was excited with 543-nm HeNe laser, and the emission was filtered using a spectral detector. To track Ca^2+^ movements across the t-system membrane, images were continuously recorded in *xyt* mode with an aspect ratio of 256 × 512, with the long aspect of the image parallel with that of the preparation. Each *xy* frame was captured in 0.8 s.

T-system rhod-5N fluorescence (*t*) [*F* (*t*)] was collected during continuous *xyt* imaging during multiple internal solution changes. Figure 1B shows an example from a WT skinned fiber with t-system–trapped rhod-5N, where the [Ca^2+^]_t-sys_ transient ([Ca^2+^]_t-sys_ (*t*)) during cytoplasmic solution exchanges was tracked to determine RyR1 Ca^2+^ leak. The [Ca^2+^]_t-sys_ (*t*) is first measured at 1.2 mM in response to standard resting solution with 67 nM [Ca^2+^]_cyto_ (values close to the expected physiological cytoplasmic and t-system [Ca^2+^] levels). A unidirectional flux was generated by depleting the SR of Ca^2+^ with caffeine (to induce SOCE), which caused a rapid decline of the [Ca^2+^]_t-sys_ to around 0.1 mM. Applying, in this example, 200 nM [Ca^2+^]_cyto_ to the Ca^2+^-depleted fiber caused a rapid rise in [Ca^2+^]_t-sys_ (*t*) to a new steady state, about 1.4 mM [Ca^2+^]_t-sys_, because the SR filled with Ca^2+^ to deactivate SOCE and Ca^2+^ filled the JS to activate t-system PMCAs. The substitution of the solution containing 200 nM [Ca^2+^]_cyto_ to one containing 200 nM [Ca^2+^]_cyto_ and the RyR1 blocker 1 mM Tet caused the [Ca^2+^]_t-sys_ (*t*) to drop by ~0.3 mM. The drop in [Ca^2+^]_t-sys_ is due to the reduced [Ca^2+^]_JS_ as the RyR1 Ca^2+^ leak is blocked.

Figure 1C shows the same protocols while tracking [Ca^2+^]_t-sys_ (*t*) in a mechanically skinned fiber from *RYR1* KI HOM. In this case, the addition of Tet caused a deeper decline of [Ca^2+^]_t-sys_ than in the WT fiber, indicative of a leakier RyR1 in the HOM than the WT fiber.

At the end of the experiment, each fiber was exposed to ionophore (ionomycin from Streptomyces conglobatus) and 5 mM Ca^2+^, followed by 0 Ca^2+^ to obtain the fluorescence maximum (*F*max) and minimum (*F*min), respectively. These values were used in conjunction with the previously determined *K*_D_ of rhod-5N in the t-system of 0.8 mM (*11*) to determine [Ca^2+^]_t-sys_, with the relationship: [Ca^2+^]_t-sys_ (*t*) = *K*_D,Ca_ * [*F* (*t*) – *F*min)/(*F*max – *F* (*t*)].

The flux of Ca^2+^ across the t-system membrane (in mM s^−1^) was determined as (*d*[Ca^2+^]/*dt*)*B_t-sys_, where *B*_t-sys_ is the Ca^2+^ buffering power of the t-system (*B*_t-sys_ = [total calcium]_t-sys_/[Ca^2+^]_t-sys_). We have previously estimated *B*_t-sys_ to be ~1 (*11*).

We have previously concluded that each application of Ca^2+^ to the fiber is independent from the others without any lasting effect (*11*, *12*). Therefore, all data collected from fibers that provided calibrations of t-system rhod-5N fluorescence and [Ca^2+^]_t-sys_ were used for analysis.

A two-way analysis of variance (ANOVA) with Tukey’s multiple comparisons was used to determine significant differences in Fig. 1F. Symbols represent # = 4 significant results and Ψ = 3 significant results. 28 nM [Ca^2+^]_cyto_: WT, HET, HOM versus CSQ1, *P* < 0.001, 0.001, 0.01, respectively. 67 nM [Ca^2+^]_cyto_: WT versus HOM and CSQ1, *P* < 0.01, 0.0001, respectively. HET versus HOM and CSQ1, *P* < 0.05, 0.0001, respectively. 200 nM [Ca^2+^]_cyto_: WT versus HOM and CSQ1, *P* < 0.0001, 0,001, respectively. HET versus both HOM and CSQ1, *P* < 0.05. 1342 nM [Ca^2+^]_cyto_: WT versus HET, HOM, and CSQ1, *P* < 0.01, 0.001, 0.001 respectively. Sex-specific leak in Fig. 1 (G to J) was compared using an unpaired *t* test with Welch’s correction, where *P < 0.05 and **P < 0.01.

### Measuring [Ca^2+^]_mito_

To measure [Ca^2+^]_mito_ in skinned fibers, preparations were incubated at 4°C for 10 min in a cytoplasmic solution containing no added Ca^2+^ and 10 μM rhod-2 acetoxymethyl (AM). The rhod-2 fluorescence from the mitochondria was monitored on the confocal microscope as the mitochondria were depolarized in a standard solution containing FCCP. The FCCP-induced rhod-2 spike was used to determine *F*max, and *F*min was determined as the lowest rhod-2 fluorescence signal achieved in the presence of FCCP (extended data fig. S9). These values were used in conjunction with the previously determined *K*_D_ of rhod-2 in the mitochondria of 1.54 μM (*24*) to determine [Ca^2+^]_mito_ with the relationship [Ca^2+^]_mito_ (*t*) = *K*_D,Ca_ * [*F* (*t*) – *F*min)/(*F*max – *F* (*t*)].

### Ex vivo muscle function testing

At 12 weeks of age, *RYR1* KI mice were anesthetized via intraperitoneal injection of sodium pentobarbitone (6 mg/kg), such that they did not respond to tactile stimuli. Isometric contractile properties of isolated fast twitch EDL were evaluated ex vivo, as described in detail previously (*47*). Briefly, EDL muscles were surgically excised and transferred to a 1305A Whole Mouse Test System (Aurora Scientific, ON, Canada) organ bath filled with Krebs Ringer solution (137 mM NaCl, 24 mM NaHCO_3_, 11 mM d-glucose, 5 mM KCl, 2 mM CaCl_2_, 1 mM NaH_2_PO_4_H_2_O, 1 mM MgSO_4_, 0.025 mM d-tubocurarine chloride), bubbled with Carbogen (5% CO_2_ in O_2_, BOC gases, Preston, Australia), and maintained at 25°C. EDL muscles were stimulated by supramaximal 0.2-ms square wave pulses of 350-ms train duration delivered by two platinum electrodes that flanked the length of the muscle. All stimulation parameters and contractile responses were controlled and measured using control and analysis software (DMC v5.415, Aurora Scientific, ON, Canada). Maximal twitch force at optimal muscle length was determined by delivering a 1-Hz stimulation pulse every 30 s with micromanipulations of muscle length in between pulses, until a plateau in the maximum peak twitch (Pt) force occurred. Following 4 min of rest, maximum isometric tetanic force (Po) production was determined from the plateau of the force frequency curve, whereby the EDL muscles were stimulated at 10, 30, 50, 60, 80, 100, and 120 Hz with 2-min rest between stimulations. Following determination of Po, fatigability of the EDL muscle was determined by the loss of muscle force following repeated submaximal stimulations (60 Hz) once every 5 s for 3 min at optimal length. Muscle cross-sectional area was determined following guidelines outlined in the standard operating procedure for measuring isometric force of isolated mouse muscles in vitro by TreatNMD (https://treat-nmd.org/) (*48*). Absolute tetanic force (Po) was normalized for muscle cross-sectional area and expressed as specific force (kN·m^−2^).

### Measuring total calcium in the skinned fiber

For force transducer experiments, the standard K-HDTA solution contained HDTA^2−^, 50 mM (Fluka, Buchs, Switzerland); total ATP, 8 mM; Na^+^, 36 mM; K^+^, 126 mM; total Mg^2+^, 8.5 mM (giving 1 mM free [Mg^2+^]); creatine phosphate, 10 mM; total EGTA, 0.075 mM; Hepes, 90 mM; pH 7.1 and pCa (−log_10_ [Ca^2+^]) ~7.1, except where stated. Where required, the SR of the skinned fiber was totally depleted of all releasable Ca^2+^ by exposure to the “full release solution,” which was similar to the K-HDTA solution but with 30 mM caffeine, 0.05 mM free Mg^2+^ (total Mg^2+^ of 2.1 mM), and 0.5 mM free EGTA (pCa 8.5) present to chelate released Ca^2+^. Where required, the SR was maximally loaded with Ca^2+^ by exposing the fiber for 4 min to a load solution, which was the same as the standard K-HDTA solution but with the pCa buffered at 6.7 with 1 mM total CaEGTA-EGTA. Under these conditions, a 4-min load period was sufficient to load the SR close to its maximum capacity [see (*15*)].

Following the procedure described (*49*) to measure the maximum force produced by the skinned fiber and to ensure that BAPTA-lysing experiments (see below) did not appreciably alter myofibrillar Ca^2+^ sensitivity, heavily Ca^2+^-buffered solutions were prepared in which all HDTA was replaced by EGTA (relaxing solution) or CaEGTA (maximum Ca^2+^ activating solution). The relaxing solution contained 50 mM EGTA and no added Ca^2+^ (pCa > 9), and the maximum Ca^2+^-activating solution (“max”) contained 49.5 mM Ca^2+^ (pCa 4.7), with total Mg^2+^ of 10.3 and 8.1 mM, respectively, to maintain the free [Mg^2+^] at 1 mM. These two solutions were mixed in appropriate ratios to produce solutions with pCa in the range 6.7 to 4.7.

The BAPTA solution used to pre-equilibrate fibers before lysing (see below) was similar to the standard K-HDTA solution but had no EGTA and instead had 0.1 to 15 mM BAPTA added from a 47 mM BAPTA stock solution. The BAPTA stock solution was titrated with Ca^2+^ using a Ca^2+^-sensitive electrode (Orion Research, Boston, MA, USA) to establish the exact amount of BAPTA present and was the same stock as used in our previous studies (*15*, *49*). In some experiments, 25 μM FCCP was present in both the full release solution and BAPTA pre-equilibration solution to abolish the mitochondrial membrane potential and release all Ca^2+^ present in the mitochondria. FCCP was added from a freshly prepared 50 mM stock solution in dimethyl sulfoxide.

#### Contractile apparatus experiments

The force-Ca^2+^ relationship was determined by exposing the skinned fiber segment to a sequence of heavily buffered solutions at progressively higher free [Ca^2+^] (50 mM CaEGTA-EGTA, pCa >9 to 4.7), with maximum force defined as that elicited at pCa 4.7. Isometric force produced at each [Ca^2+^] was expressed as a percentage of the corresponding maximum force and analyzed by fitting a Hill curve using GraphPad Prism 6 software to ascertain the pCa_50_ (pCa at half-maximum force) and Hill coefficient (*h*) for each sequence.

#### Lysing experiment to quantify total Ca^2+^ content

As described previously (*14*, *15*, *49*), the total amount of Ca^2+^ contained in a fiber can be quantified by pre-equilibrating the skinned fiber in a solution with a known concentration of the very fast calcium buffer BAPTA and then transferring the fiber to an emulsion of 10% Triton X-100 and paraffin oil (TX-oil) to lyse all membranous compartments and release any Ca^2+^ from within the fiber. Briefly, the skinned fiber was first placed in the standard weakly Ca^2+^-buffered K-HDTA solution for 2 min to wash out all the diffusible Ca^2+^-binding proteins present endogenously in the cytoplasm without altering the Ca^2+^ content of the fiber. The skinned fiber was then equilibrated for 20 s in a solution with a set [BAPTA] before being placed in a freshly triturated emulsion of TX-oil (10% v/v). The Ca^2+^ released upon the membrane lysing rapidly binds to the known amount of BAPTA present within the fiber and to other sites, predominantly troponin C (TnC). If the pre-equilibrating [BAPTA] was chosen such that the fiber produced a finite, nonmaximal force response upon lysis, then the total amount of Ca^2+^ present in the fiber could be calculated in absolute terms from the [BAPTA] in the equilibration solution and the magnitude of the force response. Other skinned fiber segments, before the TX-oil lysing, were (i) fully depleted of their endogenous SR Ca^2+^ content by a 1-min exposure to the full release solution or (ii) loaded to their maximal SR Ca^2+^ capacity by a 4-min exposure to standard load solution (pCa 6.7, buffered with 1 mM total EGTA) (see “Measuring total calcium in the skinned fiber”).

#### Calculation of Ca^2+^ release from lysing experiment

The total Ca^2+^ content within the fiber at the time of lysis ([Ca^2+^]_T_), expressed in millimoles per liter total fiber volume [in keeping with previous studies (*14*, *15*, *49*)] could be calculated as the sum of (i) the Ca^2+^ bound to BAPTA, (ii) the Ca^2+^ bound to all other high-affinity binding sites in the fiber (predominantly TnC), and (iii) the free Ca^2+^ in the myoplasm ([Ca^2+^]), as described in detail previously (*15*). Briefly, the total amount of Ca^2+^ within a given fiber ([Ca^2+^]_T_) was calculated as follows:

1. The cytoplasmic free Ca^2+^ concentration ([Ca^2+^], in molar units) within the fiber at the peak of the force response elicited upon lysis was calculated from the relationship between force and [Ca^2+^], which was defined as being the Hill curve with values of pCa50 and Hill coefficient for that fiber of the specified mouse genotype. Mean values of pCa50 and Hill coefficient used for calculation were 5.71 and 5.2, respectively, for WT fibers; 5.81 and 6.9, respectively, for CSQ1 null fibers; 5.80 and 5.9, respectively, for RyR^KI/WT^ fibers; and 5.83 and 6.1, respectively, for RyR^KI/KI^ fibers. The [Ca^2+^] was calculated analogously to equations 1 and 2 in (*15*).

2. The effective [BAPTA] within the fiber was taken as being 1.13 times the [BAPTA] of the pre-equilibration solution, to account for the swelling of the fiber when initially placed in solution, and also the fiber volume to which BAPTA was not accessible (i.e., that occupied by the SR, t-tubular system, and mitochondria) (*14*).

3. The percentage of BAPTA with bound Ca^2+^ (%CaBAPTA) was determined from the size of the force response, the relevant force-[Ca^2+^] relationship (see calculation of [Ca^2+^] above), and the known Ca^2+^-binding properties of BAPTA (*50*) [see (*39*)], calculated analogously to that set out in equations 3 and 4 in (*15*).

4. The total possible increase in Ca^2+^ binding to TnC was taken as 140 μM binding to the Ca^2+^-specific sites in EDL fast twitch fibers, and a further 140 μM binding to the non–Ca^2+^-specific sites also present in those fibers (i.e., two Ca^2+^-specific sites and two nonspecific sites per TnC molecule) (*14*). The amount of Ca^2+^ binding to TnC (CaTnC) (in millimolar) as a function of force was calculated based on the data of (*51*) and (*52*) as described previously (*15*).

5. Ca^2+^ binds to the ATP and HDTA present, and the total of these and the free [Ca^2+^] was estimated as being ~9.6 × [Ca^2+^] (*39*).

6. Last, 0.015 mM was deducted from the total to take into account the contaminating Ca^2+^ present in the BAPTA pre-equilibration solution. The total Ca^2+^ content within the fiber at the time of the lysis (expressed in millimoles per liter fiber volume) was thus calculated as being [Ca^2+^]_T_ = 1.13 × [BAPTA] × %CaBAPTA/100 + CaTnC + 9.6 × [Ca^2+^] × 1000 – 0.015.

### Western blotting

The relative abundance of calcium signaling and mitochondrial proteins in EDL muscles of WT and CSQ KO mice was analyzed using a semiquantitative Western blotting technique following procedures previously described (*53*, *54*). Proteins in WT and CSQ KO whole-muscle homogenate samples and whole-muscle homogenate mix (4- to 5-point calibration curve), alongside PageRuler Prestained Protein Ladder (Thermo Fisher Scientific, Rockford, USA), were separated on either 4 to 15% Criterion TGX Stain-Free gels or 4 to 12% Criterion bis-tris gels (Bio-Rad, Hercules, CA, USA) depending on the protein(s) of interest (*54*). Following gel electrophoresis, the total protein of each sample on a gel was visualized through ultraviolet exposure using a Criterion Stain-Free imager (Bio-Rad), and the total protein was wet transferred to a nitrocellulose membrane (100 V, 30 min). Following transfer, the membrane was rinsed with Milli-Q H_2_O and incubated in Miser Antibody Extender solution (Thermo Fisher Scientific, Rockford, IL, USA) for 10 min, rinsed five times in Milli-Q H_2_O, blocked for ~2 hours in blocking buffer [5% skim milk in 1× tris-buffered saline with Tween 20 (1× TBST)], and washed twice for ~30 s in 1× TBST. Subsequent to blocking and washing, the membrane was cut horizontally at designated molecular weights, enabling separate probing of multiple proteins of interest at variable molecular weights and minimizing the number of primary antibody reprobes for an individual membrane section. The individual membrane sections were exposed to primary antibodies diluted in 1% bovine serum albumin in phosphate-buffered saline in Tween 20 (1× PBST) and 0.02% sodium azide overnight at 4°C and 2 hours at room temperature (RT), all with constant rocking. The primary antibodies used and the assigned dilutions were RyR1 [mouse, 1:100; Developmental Studies Hybridoma Bank (DSHB), IA, USA, 34C], DHPR (mouse, 1:400; DSHB, IIID5EI), PMCA (mouse, 1:1000; Abcam, Cambridge, UK, ab2825), optic atrophy 1 (OPA1; mouse, 1:1000; BD Biosciences, San Jose, CA, USA, #612607), MFN2 (rabbit, 1:2000), dynamic-related protein 1 (DRP1; mouse, 1:1000; Cell Signaling Technology, MA, USA, #8570), oxidative phosphorylation (OXPHOS; mouse, 1:1000; Abcam, ab110411), MiD49 (rabbit, 1:500), actin (rabbit, 1:500; Sigma Aldrich, #A2066), NADH:Ubiquinone Oxidoreductase Subunit A9 (NDUFA9; rabbit, 1:1000), and cytochrome c oxidase subunit IV (COXIV; rabbit, 1:1000; Cell Signaling Technology, #4844), as described previously (*54*). The MFN2, MiD49, and NDUFA9 were gifts from M. Ryan. Following primary antibody incubation, membranes were washed three times in blocking buffer (2, 10, and 10 min), rinsed in 1× TBST, and exposed to corresponding horseradish peroxidase (HRP)–conjugated secondary antibodies diluted in blocking buffer for ~60 to 90 min at RT, all with constant rocking. The secondary antibodies used and the assigned dilutions were goat anti-mouse HRP (1:60,000; Thermo Fisher Scientific, VIC, Australia, PIE31430) and goat anti-rabbit HRP (1:60,000; Thermo Fisher Scientific, PIE31460). Following secondary antibody incubation, membrane was washed three times in 1× TBST (2, 10, and 10 min) and coated in West Femto chemiluminescent substrate (Thermo Fisher Scientific) to visualize protein bands. Molecular weight markers were exposed under white light, and chemiluminescent images were captured without moving the membrane using Chemidoc MP System and densitometry performed using Image Lab 5.2.1 (Bio-Rad). The total protein (Criterion TGX Stain-Free gels) or total actin protein (Criterion Bis-Tris gels) and protein of interest were normalized to their respective calibration curves on the same gel, and then expressed relative to the average of the WT mice on a Western blot (*53*).

#### Citrate synthase

The citrate synthase activity was measured in EDL muscle of WT (*N* = 6) and CSQ KO (*N* = 6) mice, similar to methods previously described (*51*). A reference cuvette contained 840 μl of 0.1 M tris buffer, 100 μl of 5′,5-dithiobis (2-nitrobenzoic acid) (0.5 mg ml^−1^ made in tris buffer), 10 μl of acetyl-coA (6 mg ml^−1^ made in tris buffer), and 50 μl freshly prepared oxaloacetate acid (1.2 mg ml^−1^ in tris Buffer) and absorbance set to zero at 412 nm in a spectrophotometer (SpectraMax M5e, Molecular Devices, USA). The blank cuvette was read for 3 min and at 15-s intervals during activity data collection to ensure that stable baseline readings were obtained. Sample cuvettes for all WT and CSQ KO mice were similarly prepared to the reference cuvette with the addition of 15 μl of muscle homogenate for each animal. For all blank and sample cuvettes, once the oxaloacetate acid solution was added, the citrate synthase activity readings began with absorbance recorded at 15-s intervals for 3 min. The overall change in absorbance (∆Abs) was determined, and the citrate synthase activity was presented as μmol min^−1^ total protein^−1^.

### Electron microscopy

Intact EDL muscle was dissected from WT and *RYR1* KI (HOM and HET) mice and processed for EM using a modification of the method of (*55*). As previously described (*56*), muscle was rapidly excised, immersed in a solution of 2.5% glutaraldehyde in PBS, and immediately irradiated in a Pelco Biowave (Ted Pella Inc.) for 3 min at 80 W under vacuum. Samples were transferred to a fresh solution of 2.5% glutaraldehyde in PBS and left for 30 min at RT before washing in 0.1 M cacodylate buffer. Samples were then immersed in a solution containing potassium ferricyanide (3%) and osmium tetroxide (2%) in 0.1 M cacodylate buffer for 30 min at RT, then in a filtered solution containing thiocarbohydrazide (1%) for 30 min at RT, osmium tetroxide (2%) for 30 min, then in 1% aqueous uranyl acetate for 30 min at 4°C. After a further staining step of 20 min in 0.06% lead nitrate in aspartic acid (pH 5.5) at 60°C, samples were dehydrated and embedded in Epon LX112 resin. Mitochondrial measurements [V(v)] and surface density [S(v)] were calculated using standard stereological methods on random sections as described in (*57*).

### Model of Ca^2+^ distribution

The Ca^2+^ distribution model (fig. S13) was used to determine the likely basis of the low twitch force output of *RYR1* KI HOM fibers compared to the fibers from WT and *RYR1* KI HET animals (Fig. 6). The model has been described in detail previously (*19*, *20*). Briefly, the model incorporates two compartments: the SR and cytoplasm. Ca^2+^ is released from the SR with a time course that mimics those described by (*38*). The temporal evolution of the distribution of Ca^2+^ in the cytoplasm is determined by the binding kinetics of Ca^2+^ sites on TnC, parvalbumin (Pv), ATP, and the Ca^2+^ indicator dye. Ca^2+^ is returned to the SR via the SERCA. The rate of pumping was assumed vary in a sigmoidal fashion on [Ca^2+^]_cyto_ with a slope of 2 and with half-maximal rate achieved when [Ca^2+^]_cyto_ was 50 μM (*58*). Isometric force generation was determined using a two-state cross-bridge model. The rates of cross-bridge attachment and detachment were constrained (*59*) so that they were consistent with the force-dependent rate of heat output in an isometric contraction (*60*) and the simultaneous attachment of 30% of cross-bridges during isometric contraction (*61*). The cross-bridge model incorporated Ca^2+^-dependent force generation, implemented by assuming a sigmoidal dependence of the rate of cross-bridge attachment on the extent of binding of the second Ca^2+^ to each TnC. The steady-state force-pCa relationship calculated using the model was consistent with that for mouse fast muscle (*62*) when the sigmoid describing the Ca^2+^-binding dependence had a slope of 3.5 and half maximal binding rate at 36 μM. Using these criteria, the rates of cross-bridge attachment and detachment were 170 μM s^−1^ and 396 s^−1^, respectively.

The model was also used to estimate the heat produced in the resting fiber due to Ca^2+^ cycling between the SR and cytoplasm. It was assumed that one ATP was used to pump 2 Ca^2+^ ions and that the ATP consumption was balanced by oxidative ATP generation by the mitochondria. The net heat resulting from ATP hydrolysis and its oxidative regeneration is 74 mJ (μmol ATP)^−1^ (*21*). The rate of Ca^2+^ pumping into the SR was calculated on the basis of a sigmoidal relationship (slope, 2; 50% maximum rate at 500 nM) between Ca^2+^ pumping rate and [Ca^2+^]_cyto_ and the reported values of [Ca^2+^]_cyto_ in resting fibers from each of the genotypes. The projections also rely on the *K*_M_ of SERCA for Ca^2+^ not changing between the genotypes. Figure 1 indicates that the SR loads Ca^2+^ at 28 nM [Ca^2+^]_cyto_ in each genotype, indicating that the SERCA of the mutant mice remains sensitive to low [Ca^2+^]; the leak from the SR is associated with a decline in SR Ca^2+^ content, indicating that the SERCA activity was not increased to compensate for SR Ca^2+^ loss (Fig. 2). Both these results indicate that the Km of SERCA for Ca^2+^ did not shift. Consistent with this, *RYR1*^Y524S^ mice have also been reported to show leak, no change in SERCA activity, with increases in basal ATP turnover and blood [lactate] (*62*).

### Simulations to determine Ca^2+^ distribution

To model the Ca^2+^ distribution in fibers from mice with different genotypes, it was assumed that the resting [Ca^2+^]_cyto_ values reflected the balance among the total cell Ca^2+^ content, the rate of leak of Ca^2+^ from the SR into the cytoplasm, and the rate of Ca^2+^ uptake into the SR. The total cell Ca^2+^ content was taken from the measurements in this study (Figs. 2 and 5), and the assumed rate of leak of Ca^2+^ from the SR into the cytoplasm was adjusted iteratively, using the model to calculate the cellular Ca^2+^ distribution in each iteration, until the modeled resting [Ca^2+^]_cyto_ matched the (scaled) experimental values (Table 2). When this was achieved, the model-derived SR free [Ca^2+^] also matched that reported here, further validating the model (Table 2). Once this had been achieved, the resting distribution of Ca^2+^ among the various buffers was calculated.

**Table 2.** Distribution of Ca^2+^ in resting fibers according to model. All concentrations in micromole (liters of cytoplasmic water)^−1^. It was assumed that, even in the absence of CSQ, about 30% of the amount of Ca^2+^ in the SR in WT fibers remained bound in some form in the SR.

	**WT**
[Ca^2+^]_cyto_ (μM)	50
[TnCa] (μM)	1.75
[PvCa] (μM)	222
[ATPCa] (μM)	0.2
[Ca]_SR_	910
[CsqCa]	21,180

The next simulation determined the transient changes in Ca^2+^ distribution produced by the release of a pulse of Ca^2+^ from the SR into the cytoplasm. The simulation started with the calculated resting Ca^2+^ distribution (Table 2) and was initiated by a Ca^2+^ release pulse that mimicked the stimulus-induced Ca^2+^ release in mouse muscle (*38*). The amount of Ca^2+^ released was adjusted so that the relative twitch forces matched those measured experimentally.

### Myoblasts isolation

Following procedures described previously (*63*), total skeletal muscle from fore- and hind limbs of 3- to 5-week-old mice containing either *RYR1*^WT/WT^, *RYR1*^WT/KI^, or *RYR1*^KI/KI^ were isolated and then digested with Dispase II plus Collagenase D (Sigma-Aldrich, USA) in the presence of 5 μM calcium chloride. The digested slurry was filtered through a 100-μm cell strainer, lysed using a red cell lysis buffer containing 155 mM ammonium chloride, 10 mM potassium bicarbonate, and 1 mM EDTA at pH 7.2, and then lastly filtered through a 40-μm cell strainer. Cells were suspended in Ham’s F-10 with 20% fetal bovine serum (FBS) and pen/strep (400 U/ml) and underwent at least six preplatings to produce a pure myoblast population. The pure myoblast populations were maintained on rat tail collagen–coated (2 μg/cm^2^) petri dishes in a proliferative media consisting of Ham’s F-10 with 20% FBS, pen/strep (100 U/ml), and fibroblast growth factor–β (2.5 ng/ml), in an incubator at 37°C in 95% air/5% CO_2_. The medium was changed every 2 to 3 days. To differentiate the myoblasts into myotubes, myoblasts were plated onto Matrigel-coated 96-well imaging plates; once the cells were >60% confluent, the proliferative medium was changed to a differentiation medium consisting of Dulbecco’s modified Eagle’s medium with GlutaMAX and glucose (4.5 g/liter), 2% heat-inactivated horse serum, and pen/strep (100 U/ml) (all from Gibco, UK). The medium was changed every 1 to 2 days, and myoblasts were differentiated for 4 to 5 days at which point they underwent imaging experiments.

Myotubes were loaded with 5 μM Fura-2 AM in 10% Pluronic F-127 for 15 min at 37°C and then 25 min at RT, then washed off twice before imaging in an imaging buffer (IB) consisting of NaCl, 133 mM; KCl, 5 mM; MgCl_2_, 1 mM; CaCl_2_, 2 mM; glucose, 5.5 mM; 4-(2-hydroxyethyl)-1-piperazineethanesulfonic acid (Hepes), 10 mM; pH 7.40 at 25°C. The dye was excited at its isosbestic point of 360 ± 5 nm with the emission measured at 510 ± 40 nm using 40× 1.3 NA objective on a Nikon Eclipse T2000 epifluorescence microscope at RT. Neutral density filters were used to reduce the intensity of the excitation light, thus preventing dye photobleaching and phototoxicity to the cells. Images were captured at 5 fps with a 2 × 2 bin through an intensified 12-bit digital intensified charge-coupled device (ORCA-ER, Hamamatsu, Japan) using IPLab software (BD Biosciences, USA). The recorded images were then imported into FIJI software using the IPLab plugin (Wayne Rasband, NIH, USA). Regions of interest were drawn within individual cells, and a time series analyzer plugin (Balaji J, Department of Neurobiology, UCLA, USA) was used to determine the temporal changes in the fluorescence intensities. These data were exported to Prism 7 (GraphPad, USA), which was used to fit linear regression models for the basal signal in IB, and then independently in Mn^2+^-containing solutions [manganese buffer (MnB)] in the absence and presence of 1 mM Tet. The MnB was similar to IB except 0.5 mM MnCl_2_ was used instead of the CaCl_2_ and MgCl_2_. The specific rate of Fura-2 quenching induced by Mn^2+^ entry was calculated by subtracting the basal slope from the slope during the MnB application (i.e., net slope) and expressed as arbitrary fluorescence units per second. The net slope was calculated in the absence and presence of Tet. Cells with a positive baseline net slope or an unstable quench following the addition of MnB were excluded from the analysis.

Nonnormally distributed data are presented as box plots showing all the data points, median, and interquartile range, with the whiskers representing the range. Statistical analyses were performed with GraphPad Prism 8 using either one-way ANOVA with a test for linear trend on data that were transformed using a log-normal transformation of the absolute values, or the Kruskal-Wallis test with Dunn’s correction for multiple comparisons. Significance was accepted as *P* < 0.05.

## Notes

View/request a protocol for this paper from *Bio-protocol*.
