# Supplementary material for: Ryanodine receptor leak triggers fiber Ca2+ redistribution to preserve force and elevate basal metabolism in skeletal muscle
Source: Sci Adv. 2021 Oct 27;7(44):eabi7166. doi: 10.1126/sciadv.abi7166 (PMC8550231; doi:10.1126/sciadv.abi7166)
Supplement: Supplementary file 1 — Figs. S1 to S13 [file sciadv.abi7166_sm.pdf]

Science Advances
AAAS

Supplementary Materials for

Ryanodine receptor leak triggers fiber Ca^{2+} redistribution to preserve force and elevate basal metabolism in skeletal muscle

Cedric R. Lamboley, Luke Pearce, Crystal Seng, Aldo Meizoso-Huesca, Daniel P. Singh, Barnaby P. Frankish, Vikas Kaura, Harriet P. Lo, Charles Ferguson, Paul D. Allen, Philip M. Hopkins, Robert G. Parton, Robyn M. Murphy, Chris van der Poel, Christopher J. Barclay, Bradley S. Launikonis*

*Corresponding author. Email: b.launikonis@uq.edu.au

Published 27 October 2021, *Sci. Adv.* **7**, eabi7166 (2021)
DOI: 10.1126/sciadv.abi7166

This PDF file includes:

Figs. S1 to S13

a

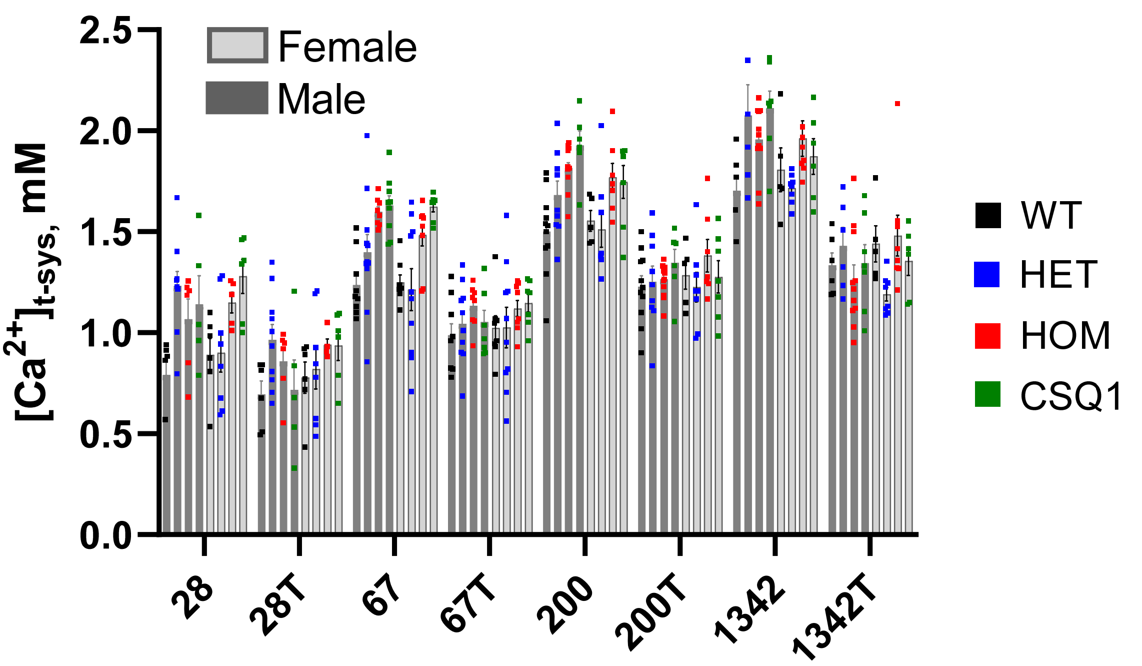

b

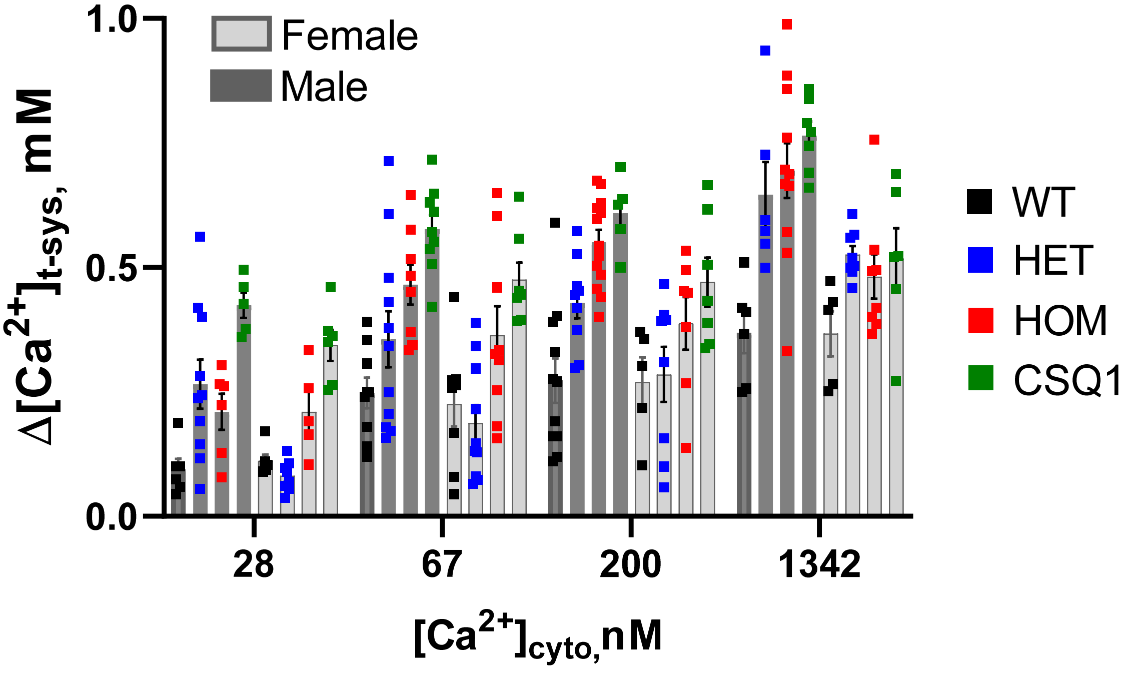

Fig S1. Steady state $[Ca^{2+}]_{t\text{-}sys}$ in the presence and absence of tetracaine (T). a, individual data points for male and female mice of each genotype. **b,** $\Delta[Ca^{2+}]_{t\text{-}sys}$ for male and female mice of each genotype ($[Ca^{2+}]_{t\text{-}sys}$ in absence of T - ($[Ca^{2+}]_{t\text{-}sys}$ in presence of T).

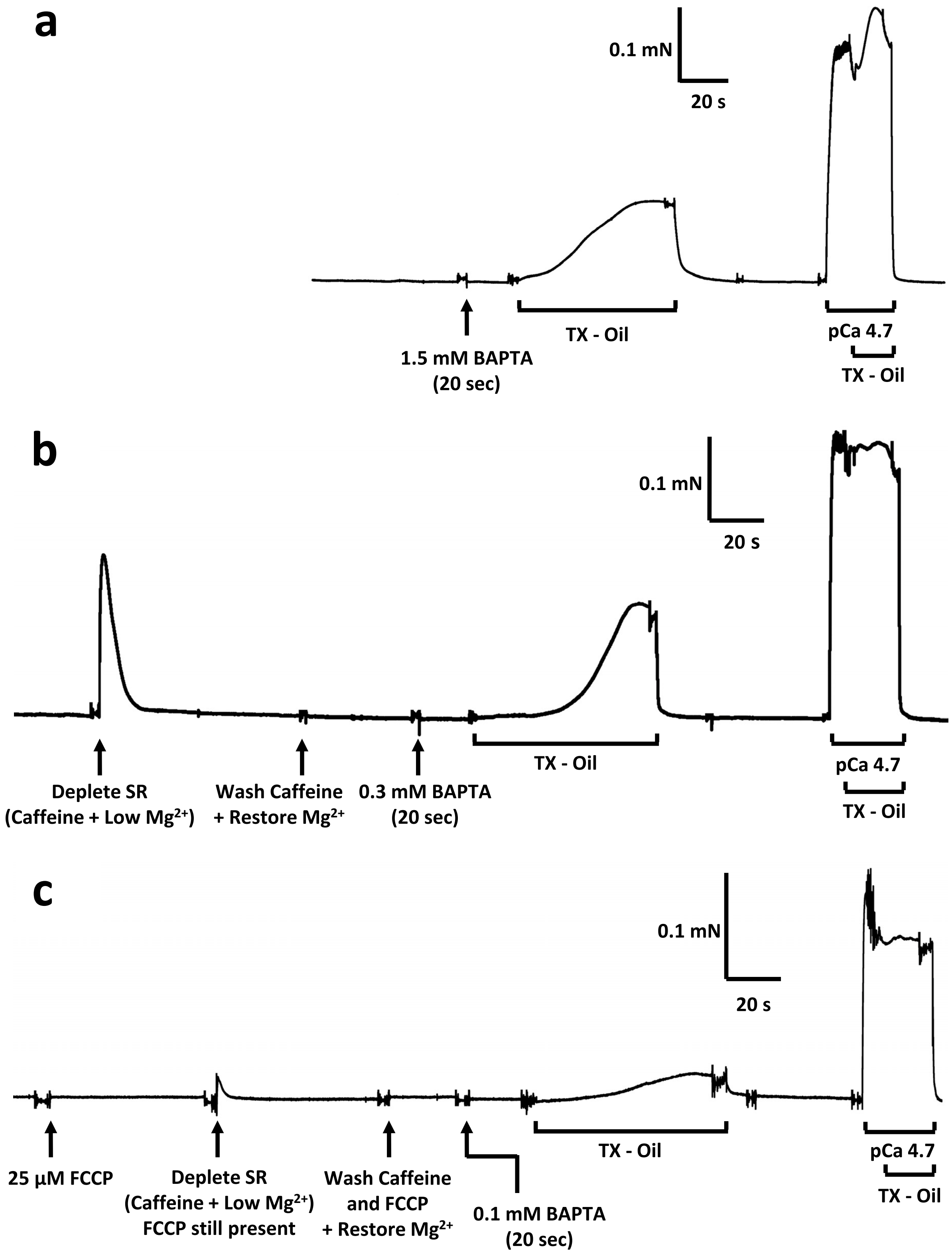

Fig S2 Force traces used to determine Ca^{2+} content. a, a skinned fibre segment from a WT mouse with endogenous resting Ca^{2+} content was briefly equilibrated with 1.5 mM free BAPTA and when lysed in the Triton-paraffin oil emulsion (TX-Oil) produced ~30% of maximal force. **b**, a skinned fibre segment from a HET RYR1 KI mouse that had been emptied of SR Ca^{2+} (by exposure to caffeine-low [Mg^{2+}] solution) and then equilibrated in 0.3 mM BAPTA produced ~41% of the maximal force response upon lysis in TX-Oil. **c**, a skinned fibre segment from a HOM RYR1 KI mouse in which the mitochondria and SR were depleted (by FCCP and caffeine-low [Mg^{2+}] exposure) required equilibration with only 0.1 mM BAPTA in order to produce ~15% of the maximal force when lysed in TX-Oil.

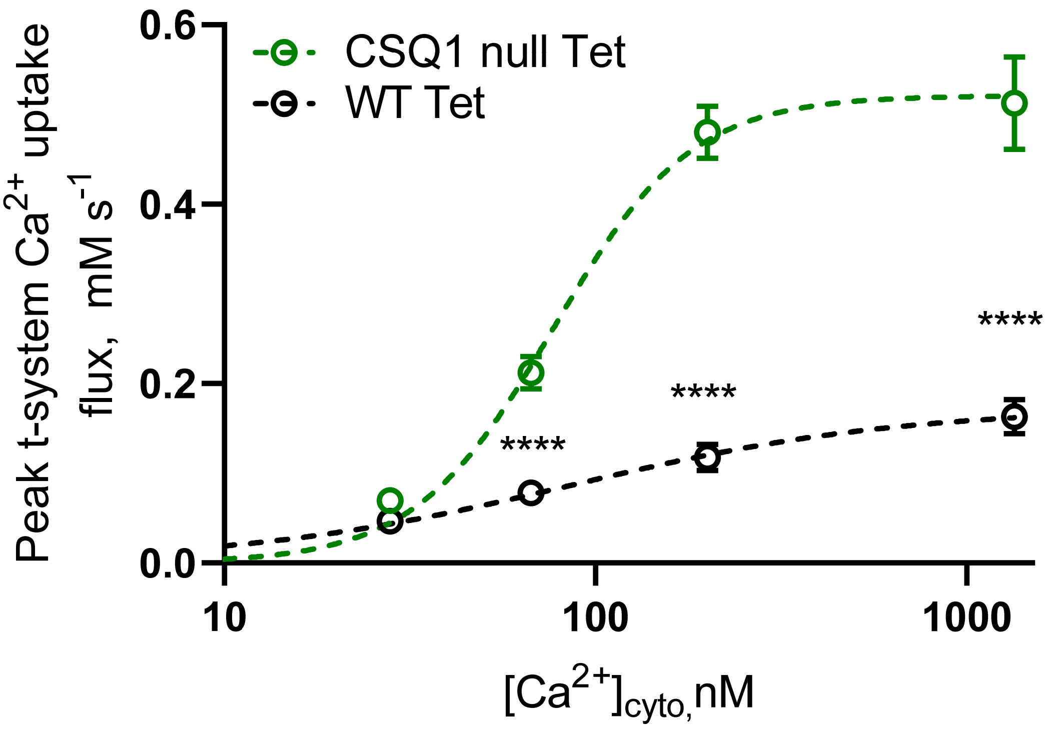

Fig S3 The peak t-system Ca^{2+} uptake rates of CSQ null fibres are increased compared to WT in the absence of RyR1 Ca^{2+} leak. Summary of peak t-system Ca^{2+} uptake rate in WT, and CSQ1 null mouse fibres with 1 mM tetracaine (Tet). Under these conditions, $[Ca^{2+}]_{JS} = [Ca^{2+}]_{cyto}$. The increase in capacity of the t-system to extrude Ca^{2+} in CSQ1 null fibres compared to WT indicates a change in $k_{D,Ca}$ in PMCA. Unpaired t-tests with Welch's corrections: ****$p<0.0001$.

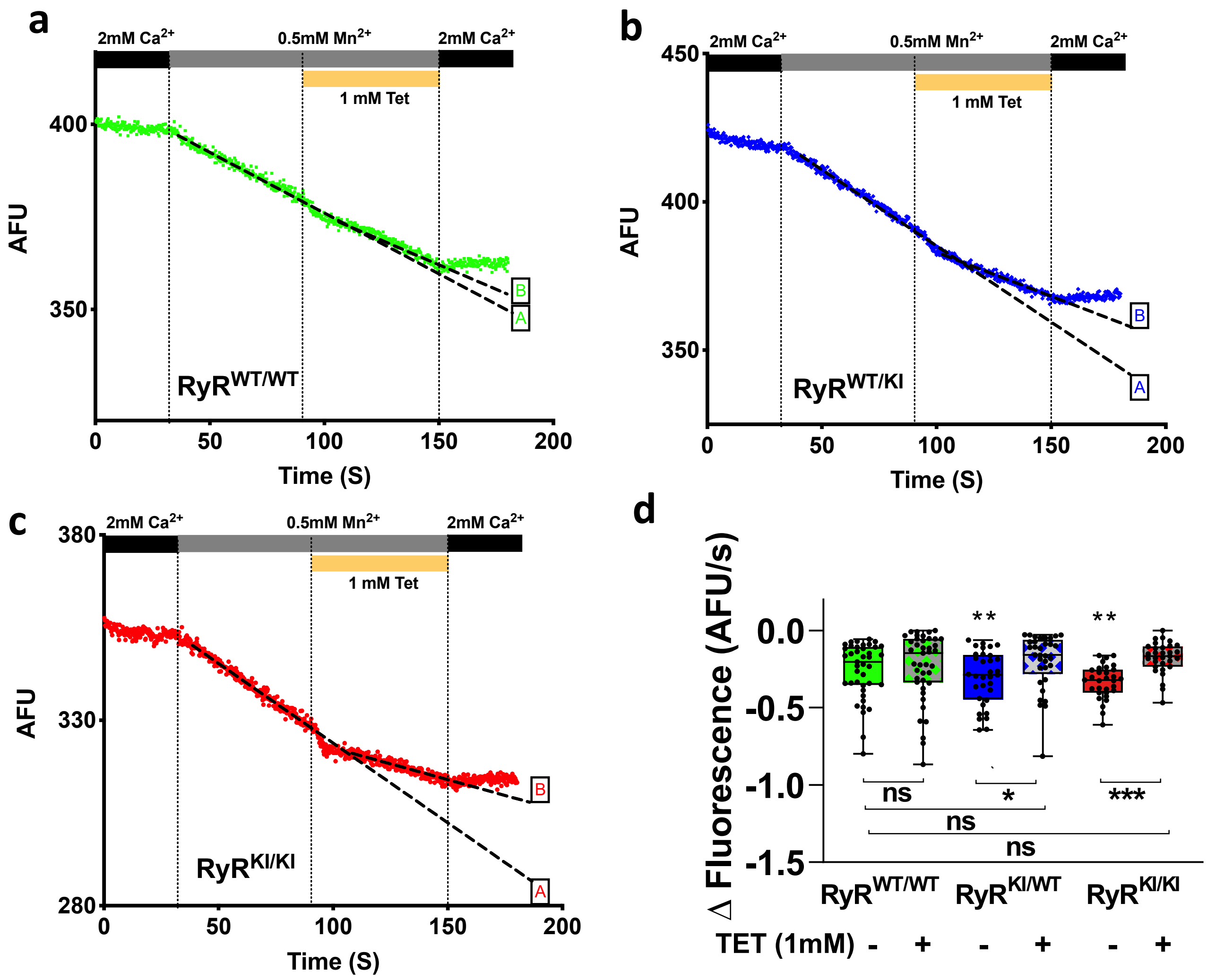

Fig S4 The cytoplasmic fura-2 fluorescence transient (excited at the isosbestic point) from myotubes derived from WT (**a**), HET (**b**) and HOM (**c**) RyR KI muscle during exposure to Mn^{2+} and then exposure to tetracaine. The dashed lines marked A and B show the changes in gradient of the quench in the presence and absence of Tet in a-c. **d**, summary of the rate of fura-2 fluorescence quench by Mn^{2+} in the presence and absence of tetracaine (Tet). In the absence of tetracaine there was a significant "gene dose"-dependent effect of RyR1 genotype on the baseline Mn^{2+} entry (one-way ANOVA with a test for linear trend, P=0.0028). In $RyR^{WT/WT}$ cells, tetracaine reduced the rate of quench from -0.203 (IQR -0.107 to -0.353) AFU/s to -0.147 (-0.056 to -0.338) AFU/s, but this was not statistically significant (P>0.999, n=41, Kruskal-Wallis with Dunn's multiple comparisons test). In $RyR^{WT/KI}$, 1 mM Tet reduced the rate of Mn^{2+} quench from -0.288 (-0.159 to -0.447) AFU/s to -0.159 (IQR -0.062 to -0.282) AFU/s (P=0.022, n=33, Kruskal-Wallis with Dunn's multiple comparisons test). Tet also reduced the rate of Mn^{2+} quench in $RyR^{KI/KI}$ from -0.320 (-0.255 to -0.403) AFU/s to -0.167 (-0.104 to -0.235) AFU/s (P=0.0004, n=31, Kruskal-Wallis with Dunn's multiple comparisons test).

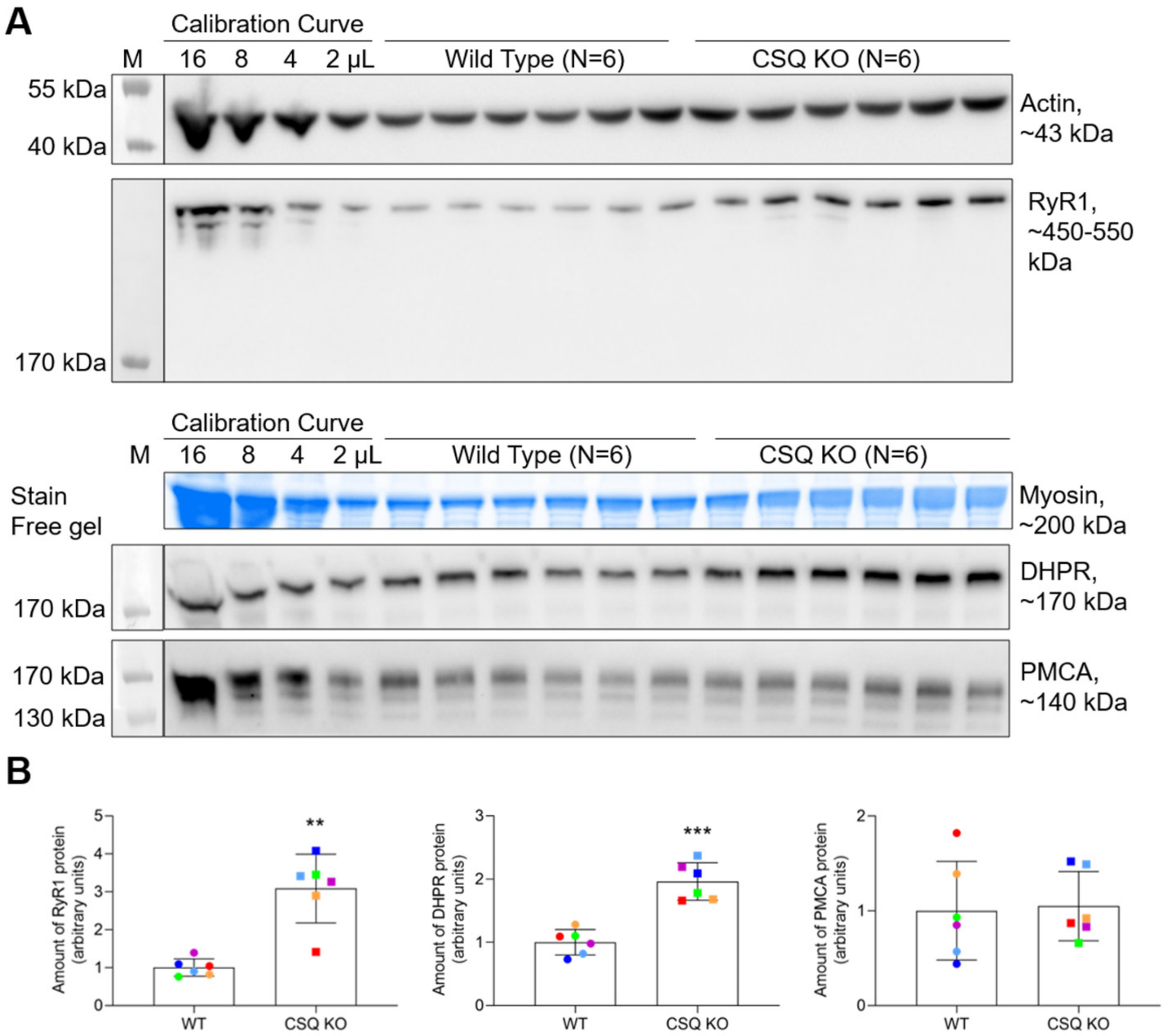

Figure S5. Calcium-signalling protein abundances in extensor digitorum longus muscle from Wild type and CSQ KO mice. a. Whole muscle homogenates of extensor digitorum longus (EDL) muscle from Wild type (WT, N=6) and CSQ KO (N=6) mice were examined for abundance of calcium-signalling proteins RyR1 (~450-550 kDa), DHPR (~170 kDa) and PMCA (~140 kDa) by Western blotting. Upper panels show Actin protein (~43 kDa) on a Bis-Tris gel and pre-transfer UV exposure of myosin protein (~200 kDa) on a Stain-Free gel, indicative of total protein in each lane/sample. Lower panels show RyR1, DHPR and PMCA protein abundances in WT and CSQ KO mice, together with a four-point whole muscle calibration curve on the same Western blot. **b.** Pooled data for relative RyR1, DHPR and PMCA protein abundances in WT (circle symbols) and CSQ KO (square symbols) mice, with each different colour indicating an individual animal which remain the same across Figs S5 and S8. Columns and horizontal bars indicate mean ± SD, respectively. For protein abundance quantification, each data point was normalised to the calibration curve on a given gel and the given total protein amount within a sample and expressed relative to the average of WT animals on the same gel (i.e. WT=1.0). To compare protein abundances between WT and CSQ KO animals and determine significance, two-tailed Welch's t-tests (unpaired t-test) were used (**p < 0.001 and p*** < 0.0001).

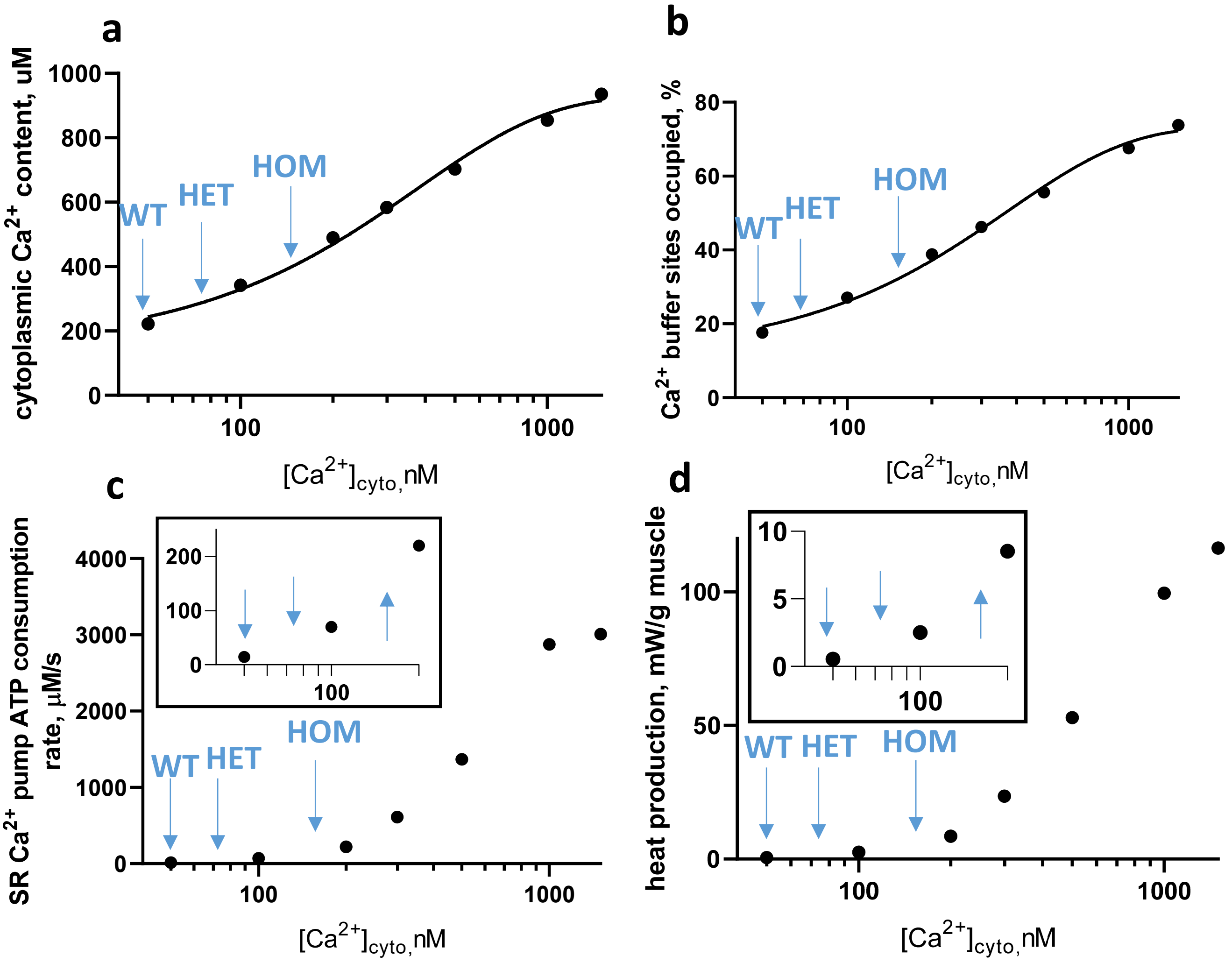

Fig S6: Cytoplasmic calcium, ATP use and heat generation. a, estimation of total Ca^{2+} bound to cytoplasmic buffers to account for resting $[Ca^{2+}]_{cyto}$ values in WT, HET and HOM fibres (cytoplasmic Ca^{2+} binding sites: parvalbumin; troponin C with one Ca^{2+} ion bound; troponin Ca with two Ca^{2+} ions bound; and ATP). **b,** % saturation of cytoplasmic Ca^{2+}-binding sites in WT and mutant mouse muscle. **c**, SR Ca^{2+} pump ATP consumption rate for a range of $[Ca^{2+}]_{cyto}$. *Inset*, expanded resting $[Ca^{2+}]_{cyto}$ range. **d**, rate of heat output in skeletal muscle as resting $[Ca^{2+}]_{cyto}$ rises. *Inset*, expanded resting $[Ca^{2+}]_{cyto}$ range. The blue arrows on each graph represent the resting $[Ca^{2+}]_{cyto}$ in intact muscle fibres.

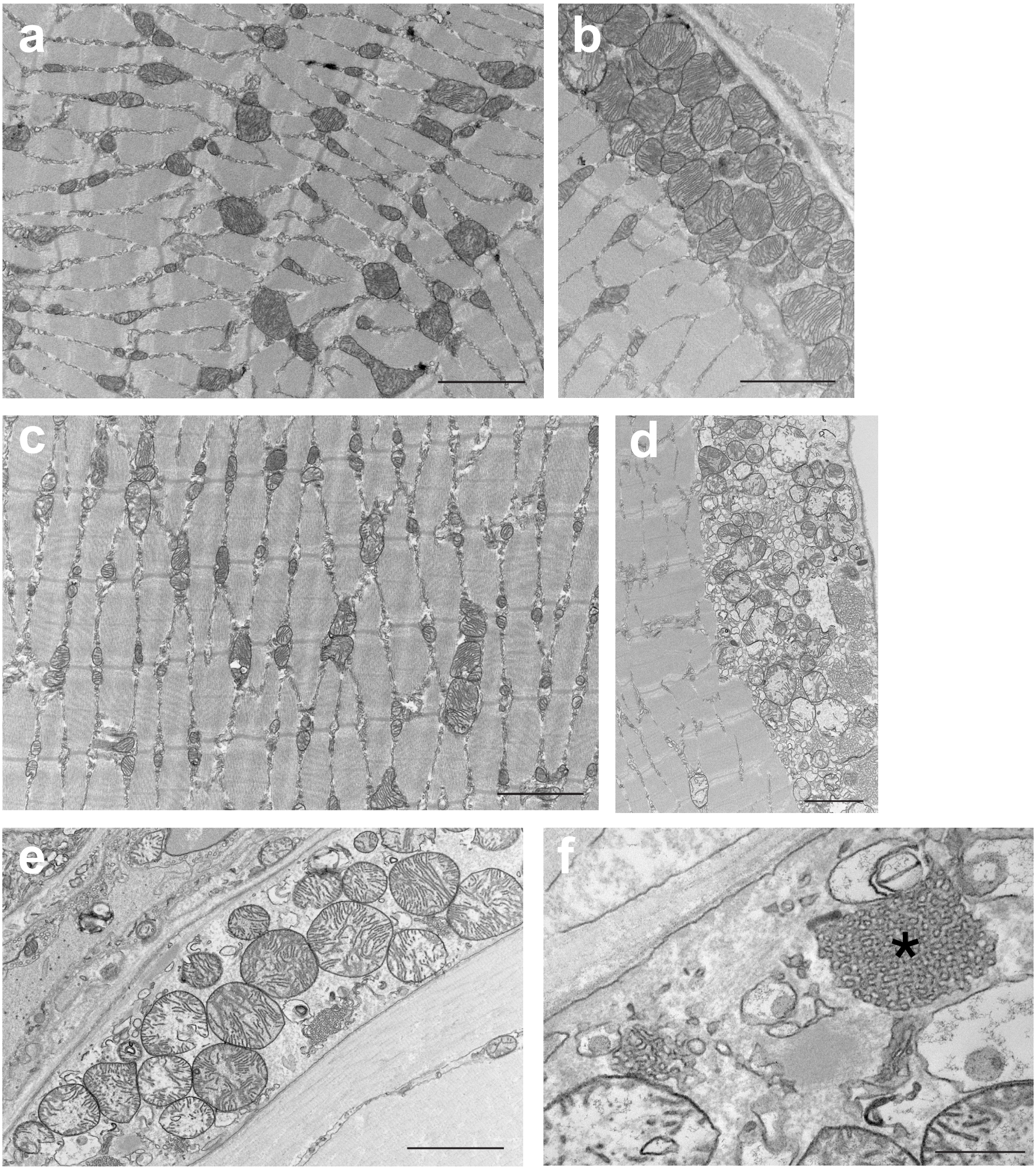

Fig S7. Structure of *RYR1* KI muscle. Normal sarcomeric and triad structure in WT (a, b) and HET (c). Circular mitochondrial morphology in HOM (d, e, f). Reticular membrane elements (highlighted by asterisk) were also observed in f. Scale bars: a-e, 2 µm; f, 500 nm.

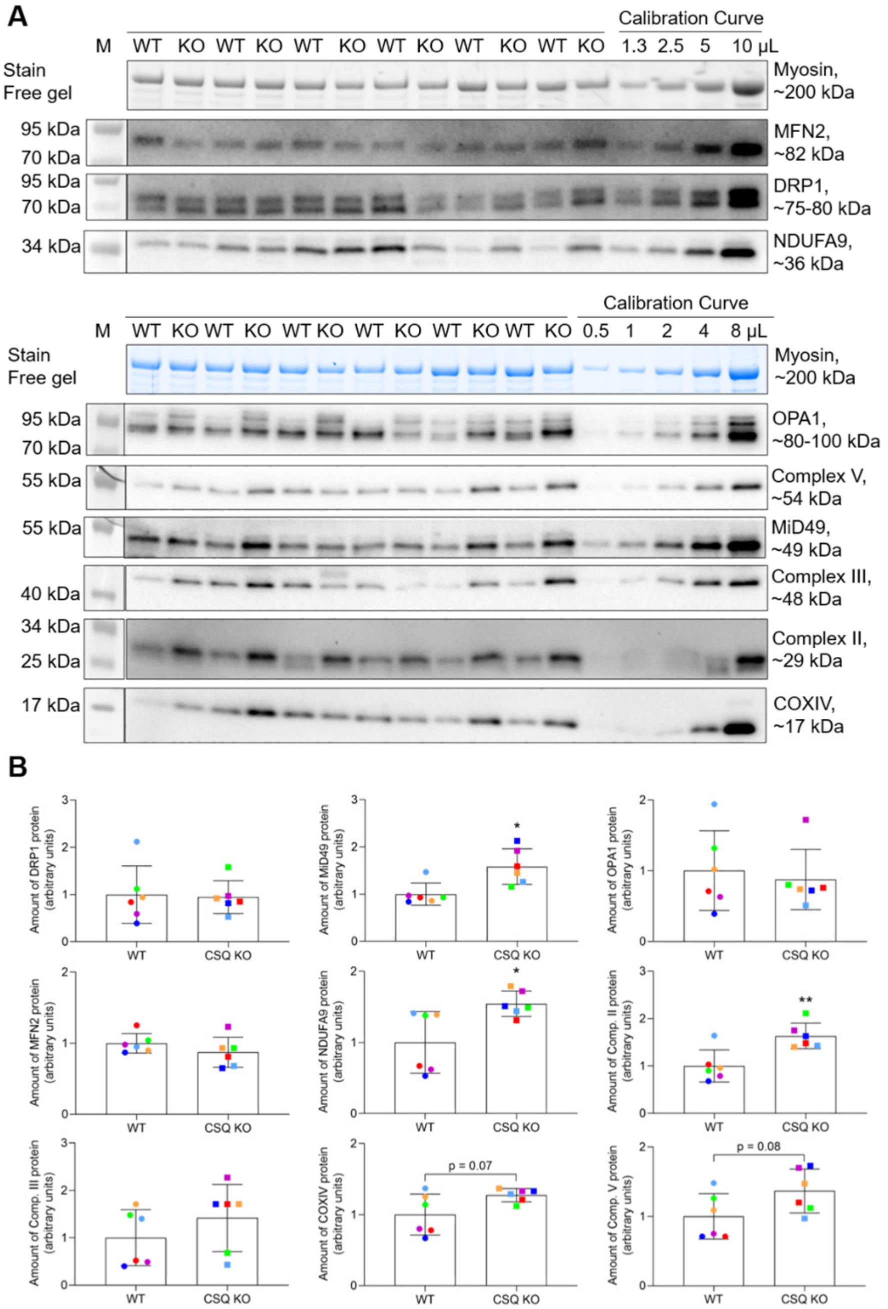

Figure S8. Mitochondrial protein abundances in extensor digitorum longus muscle from Wild type and CSQ KO mice. A. Whole muscle homogenates of extensor digitorum longus (EDL) muscle from Wild type (WT, N=6) and CSQ KO (N=6) mice were examined for abundance of calcium-signalling proteins OPA1 (~80-100 kDa), MFN2 (~82 kDa), drp1 (~75-80 kDa), Complex V (~54 kDa), MiD49 (~49 kDa), Complex III (~48 kDa), NDUFA9 (~36 kDa), Complex II (~29 kDa) and COXIV (~17 kDa) by Western blotting. Upper panels show pre-transfer UV exposure of myosin protein (~200 kDa) on different Stain-Free gels, indicative of total protein in each lane/sample. Lower panels show OPA1, DRP1, NDUFA9, OPA1, Complex V, MiD49, Complex III, Complex II and COXIV protein abundances in WT and CSQ KO mice, together with a four- or five-point whole muscle calibration curve on the same Western blot. **B.** Pooled data for relative DRP1 and MiD49 (fission proteins), OPA1 and MFN2 (fusion proteins), and NDUFA9 (Complex I), Complex II, Complex III, COXIV (Complex IV) and Complex V (abundance/content proteins) protein abundances in WT (circle symbols) and CSQ KO (square symbols) mice, with each different colour indicating an individual animal which remain the same across Figs S5 and S8. Columns and horizontal bars indicate mean ± SD, respectively. For protein abundance quantification, each data point was normalised to the calibration curve on a given gel and the given total protein amount within a sample and expressed relative to the average of WT animals on the same gel (i.e. WT=1.0). To compare protein abundances between WT and CSQ KO animals and determine significance, two-tailed Welch's t-tests (unpaired t-test) were used for DRP1, MFN2, Complex II, COXIV and Complex V proteins, and two-tailed Mann-Whitney tests were used for OPA1, MiD49, NDUFA9 and Complex III, as those data were not normally distributed (*$p < 0.05$).

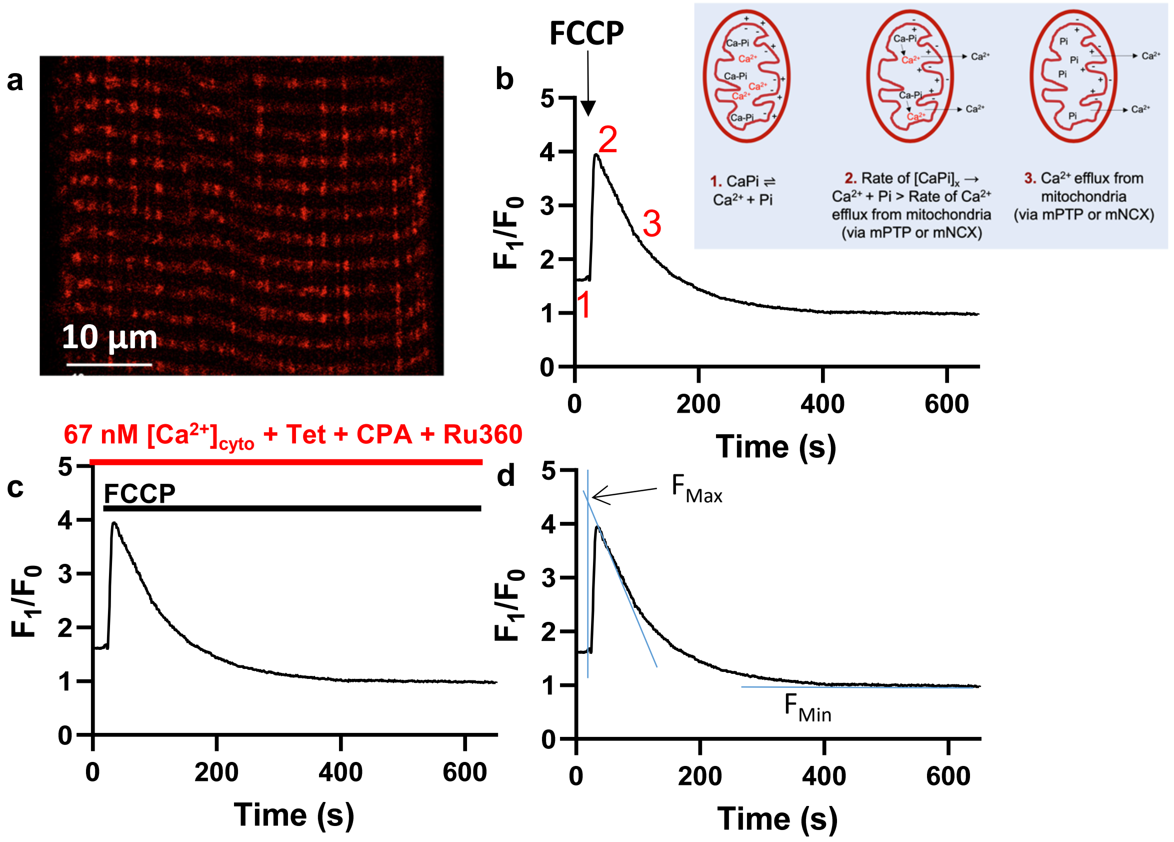

Fig. S9 Determination of F_{max} and F_{min} of rhod-2 in mitochondria of skinned fibres. a rhod-2 loaded in the mitochondria of a skinned EDL fibre. **b** rhod-2 fluorescence transient during the application of FCCP in the presence of 50 mM EGTA and 67 nM $[Ca^{2+}]_{cyto}$. A spike in $[Ca^{2+}]_{mito}$ is observed immediately after the depolarization of the mitochondrial potential. Proposed theory of FCCP-induced mitochondrial Ca^{2+} spikes: 1. Ca^{2+} is buffered in a poly-phosphate network that is dependent on the total calcium driven into the mitochondria by the potential. 2. FCCP-depolarization causes free Ca^{2+} to start leaving the mitochondria, disrupting the Ca-Pi network, causing free Ca^{2+} to spike because the rate of Ca^{2+} exit from the mitochondria is slower than the rate Ca^{2+} is released from CaPi. 3. The slow efflux of Ca^{2+} from the mitochondria occurs across the depolarized membrane. **c** Mitochondrial Ca^{2+} spike occurs in the presence of RyR, SR Ca^{2+} pump and MCU blockers, confirming mitochondrial origin of the Ca^{2+} spike. **d** the release of Ca^{2+} from the CaPi network is expected to generate an increase in $[Ca^{2+}]_{mito}$ in at least the tens of µM range, which will saturate rhod-2. To account for the slow extrusion of Ca^{2+} through the mitochondrial NCX we can back calculate where rhod-2 must be saturated: at the point FCCP is added (intersection of blue lines) to determine F_{max}. F_{min} is the point where the rhod-2 fluorescence signal reaches its minimum in the presence of FCCP. **e** following FCCP spike, the ability of the mitochondria to handle Ca^{2+} is severely impaired, suggesting that high µM levels of $[Ca^{2+}]_{mito}$ were present to open the mPTP.

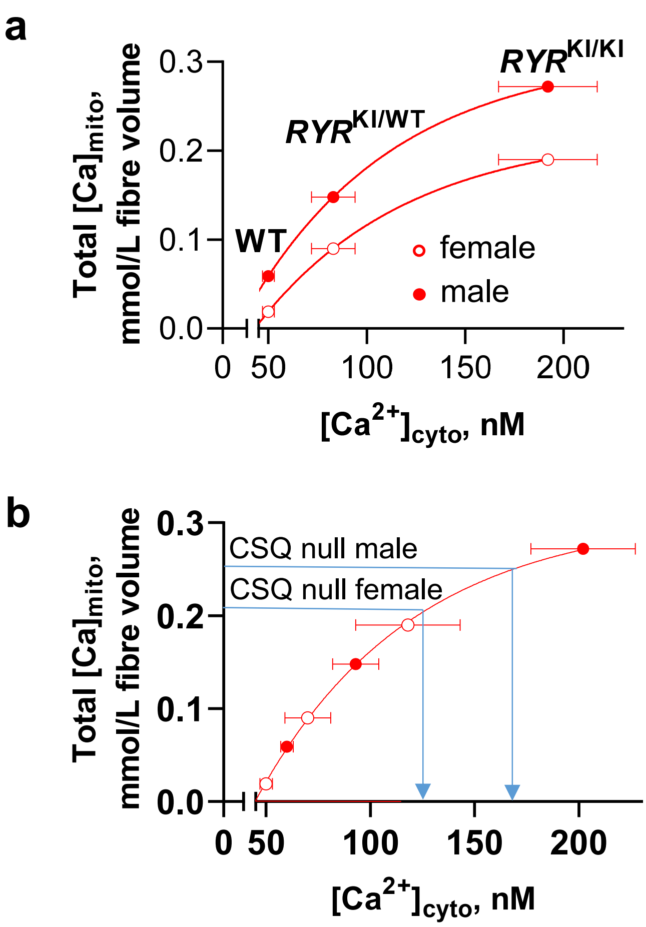

Fig S10: The relationship between $[Ca^{2+}]_{cyto}$ and total calcium content in the mitochondria. a, the $[Ca^{2+}]_{cyto}$ determined in intact muscle fibres of *RYR* KI mice (*5*) were normalized to a value of 50 nM in the WT (*28*, *29*, *30*). The total mitochondrial calcium content from males and female RYR KI mice are plotted using the same $[Ca^{2+}]_{cyto}$ (with SEM) because there was no separation of this value by sex (*5*). **b**, the greater RyR leak, lower SR calcium content and raised mitochondrial calcium content in female *RYR* KI mice compared to females means the $[Ca^{2+}]_{cyto}$ should be lower in female than male. To combine the two functions in A, we shifted the female curve to the left, to meet the male curve. This manoeuvre lowers the $[Ca^{2+}]_{cyto}$ in female, setting the WT for females at 50 nM. From this single curve the unknown $[Ca^{2+}]_{cyto}$ values in CSQ null male and female muscle fibres can be estimated using their mitochondrial calcium content (blue lines).

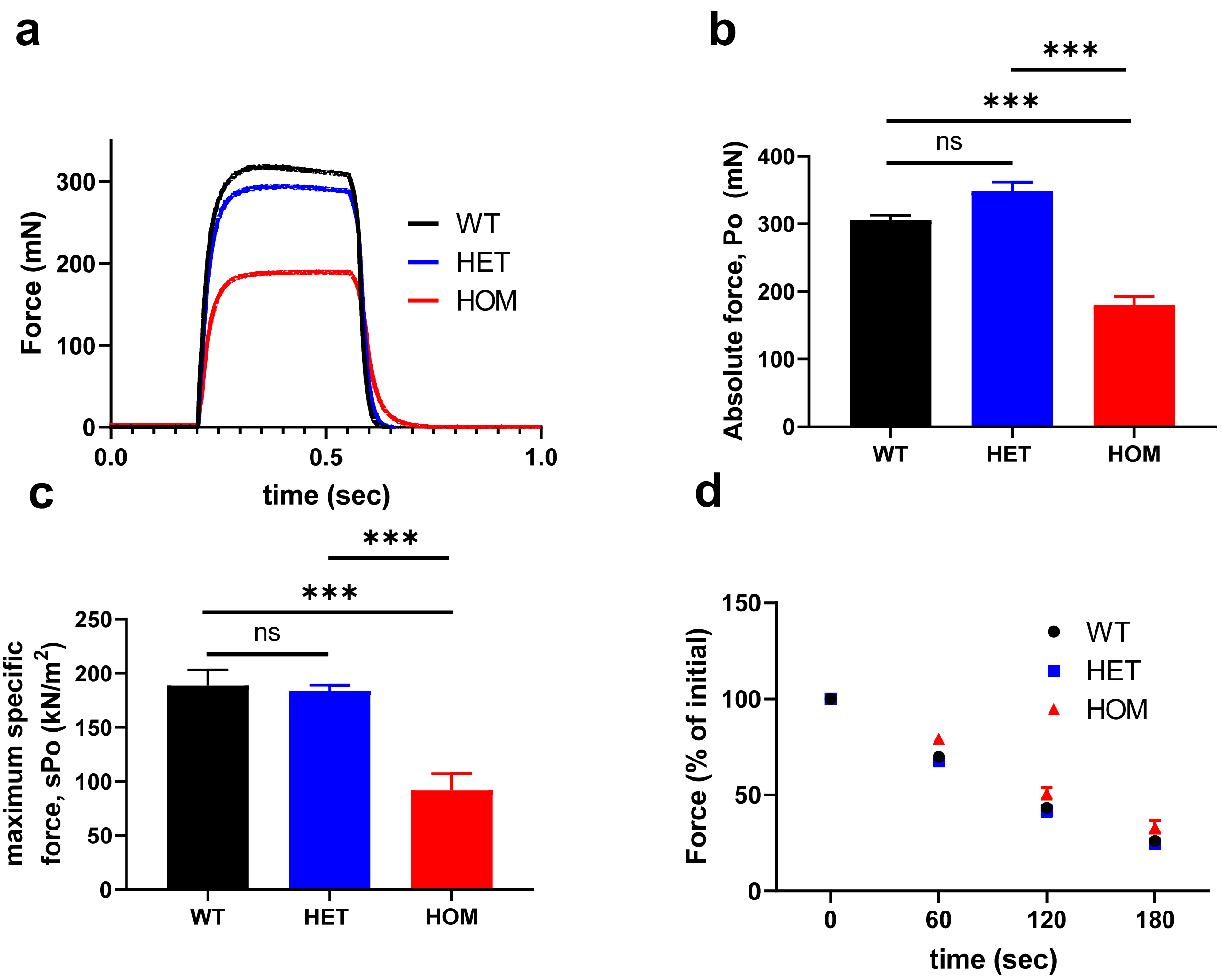

Fig S11. Force is reduced in HOM RYR1 KI muscle but not HET muscle. a examples of tetanic force responses under 120 Hz stimulation in RYR1 KI muscles. **b** absolute force (at 120 Hz stimulation) in RYR1 KI muscles. **c** maximum specific force in RYR1 KI muscles. **d** muscle stimulated every 5 s at 60 Hz for 3 min to assess fatigue susceptibility (selected data). One-way ANOVA with multiple comparisons were run in b and c: ns, not significant; ***p < 0.001. Results from 4 (WT), 7 (HET) and 4 (HOM) muscles.

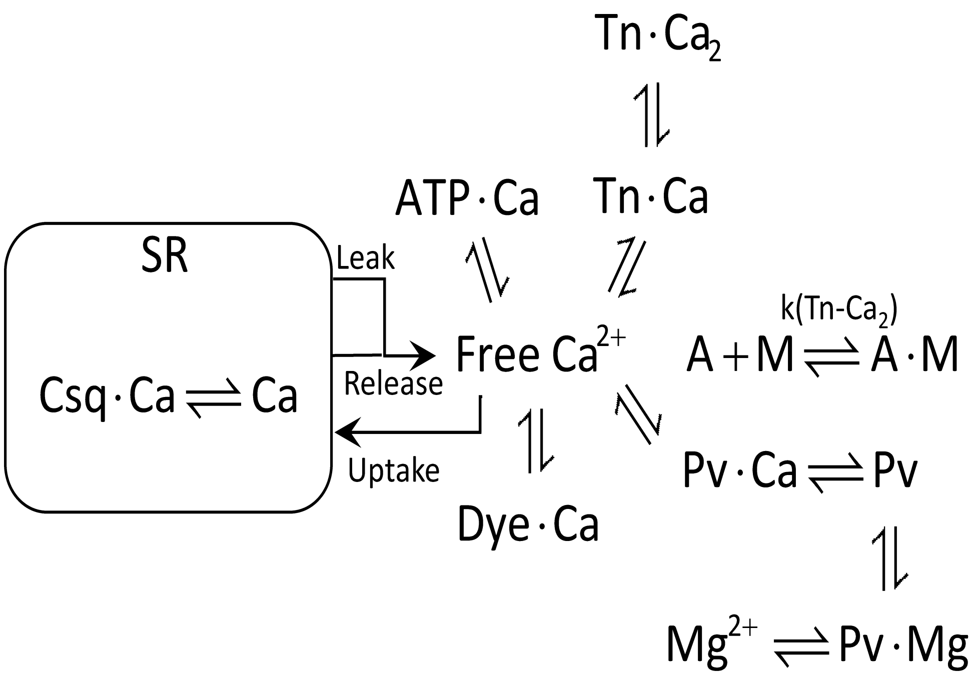

Fig S12. Schematic of the Ca^{2+} distribution model. The model was designed to describe the movement of Ca^{2+} between the SR and the cytoplasm and its binding to intracellular buffers. Tn, troponin C; Pv, parvalbumin; Csq, calsequestrin; A, actin; M, myosin. Values of rate constants and model validation in (*21*).

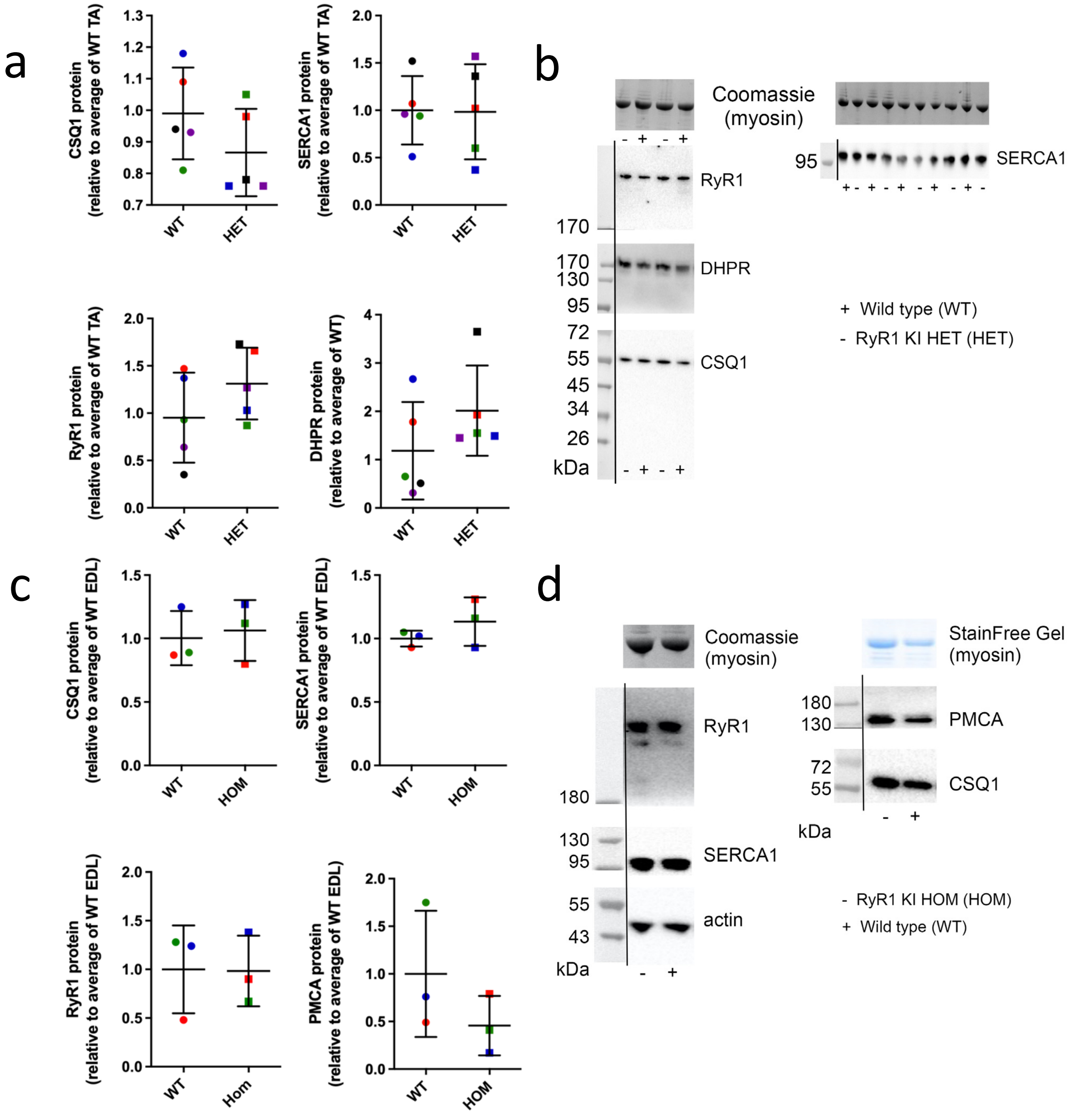

Fig S13. Calcium-handling protein abundances in muscle from WT and *RYR1* KI HOM and HET mice. A. Whole muscle homogenates of extensor digitorum longus (A and B) or tibialis anterior (C and D) muscles from WT (N=1-5, +) and *RYR1* KI (HET (-), n=5 or HOM (-), n=2-3) mice were examined for abundance of calcium-signalling proteins RyR1 (~450-550 kDa), DHPR (~170 kDa), PMCA (~140 kDa), SERCA1 (~110 kDa)),CSQ1 (~63 kDa) and/or actin (~43 kDa) by Western blotting. B and D upper panels show myosin as indicator of total protein on either 4-12% Bis-Tris gel or 4-15% Stain Free gel. Columns and horizontal bars indicate mean ± SD, respectively. For protein abundance quantification, each data point was normalised to the calibration curve on a given gel. Protein abundances between WT and HET or WT and HOM animals were compared using two-tailed unpaired t-tests.
